# Investigating the Potential Anti-SARS-CoV-2 and Anti-MERS-CoV Activities of Yellow Necklacepod among Three Selected Medicinal Plants: Extraction, Isolation, Identification, In Vitro, Modes of Action, and Molecular Docking Studies

**DOI:** 10.3390/metabo12111109

**Published:** 2022-11-13

**Authors:** Howaida I. Abd-Alla, Omnia Kutkat, Heba-tollah M. Sweelam, Wagdy M. Eldehna, Marwa A. Mostafa, Magda T. Ibrahim, Yassmin Moatasim, Mohamed GabAllah, Ahmed A. Al-Karmalawy

**Affiliations:** 1 Chemistry of Natural Compounds Department, Pharmaceutical and Drug Industries Research Institute, National Research Centre, Dokki-Giza 12622, Egypt; 2 The Center of Scientific Excellence for Influenza Viruses, Water Pollution Research Department, Environmental Research Institute, National Research Centre, Dokki-Giza 12622, Egypt; 3 Department of Pharmaceutical Chemistry, Faculty of Pharmacy, Kafrelsheikh University, Kafrelsheikh 33516, Egypt; 4 School of Biotechnology, Badr University in Cairo, Badr City 11829, Egypt; 5 Bab El-Shaariya Hospital, Al-Azhar University, Cairo 11558, Egypt; 6 Department of Pharmacognosy, Faculty of Pharmacy (Girls), Al-Azhar University, Cairo 11754, Egypt; 7 Pharmaceutical Chemistry Department, Faculty of Pharmacy, Ahram Canadian University, 6th of October City, Giza 12566, Egypt

**Keywords:** coronavirus, anti-MERS-CoV, anti-SARS-CoV-2, *Sophora tomentosa*, extraction, isolation, mode of action, molecular docking

## Abstract

The anti-MERS-CoV activities of three medicinal plants (*Azadirachta indica*, *Artemisia judaica*, and *Sophora tomentosa*) were evaluated. The highest viral inhibition percentage (96%) was recorded for *S. tomentosa*. Moreover, the mode of action for both *S. tomentosa* and *A. judaica* showed 99.5% and 92% inhibition, respectively, with virucidal as the main mode of action. Furthermore, the anti-MERS-CoV and anti-SARS-CoV-2 activities of *S. tomentosa* were measured. Notably, the anti-SARS-CoV-2 activity of *S. tomentosa* was very high (100%) and anti-MERS-CoV inhibition was slightly lower (96%). Therefore, the phytochemical investigation of the very promising *S. tomentosa* L. led to the isolation and structural identification of nine compounds (**1**–**9**). Then, both the CC_50_ and IC_50_ values for the isolated compounds against SARS-CoV-2 were measured. Compound **4** (genistein 4’-methyl ether) achieved superior anti-SARS-CoV-2 activity with an IC_50_ value of 2.13 µm. Interestingly, the mode of action of *S. tomentosa* against SARS-CoV-2 showed that both virucidal and adsorption mechanisms were very effective. Additionally, the IC_50_ values of *S. tomentosa* against SARS-CoV-2 and MERS-CoV were found to be 1.01 and 3.11 µg/mL, respectively. In addition, all the isolated compounds were subjected to two separate molecular docking studies against the spike (S) and main protease (M^pr^°) receptors of SARS-CoV-2.

## 1. Introduction

Coronavirus disease 2019 (COVID-19) is defined as an illness and the outbreak of the severe acute respiratory syndrome coronavirus 2 (SARS-CoV-2) started in Wuhan, China, spread to several other countries, and now is in its exponential phase of spread [1,2]. The most recent outbreaks of SARS-CoV and the Middle East respiratory syndrome-related coronavirus (MERS-CoV) happened in China and Saudi Arabia, respectively [3,4]. The ongoing coronavirus disease 2019 (COVID-19) pandemic has created an alarming situation for global health security. Coronaviruses (CoVs) are emerging, rapidly evolving situations, and are responsible for a growing economic, social, and mortality burden [5]. These viruses cause acute and chronic respiratory, enteric, and central nervous system diseases in humans. CoVs are known to cause various lethal respiratory infectious diseases in humans, such as SARS, MERS, and the very recent COVID-19 outbreak [6]. 

A search for new anticorona viral drugs was motivated by the emergence of MERS-CoV and the outbreak of novel SARS-CoV-2. The high virulence of these viruses and the absence of effective therapies have posed an ongoing threat to public health [7,8]. It is urgent to develop some antiviral agents for the treatment of MERS-CoV and SARS-CoV-2 infections because of their high infectivity and morbidity, besides their ability to cause epidemics worldwide [9]. The lack of an effective control strategy causes a huge risk of CoVs as these viruses have a high tendency to evolve and recombine to form new forms of viruses with the previous uncontrolled ones [10,11]. 

Great efforts have been made by researchers to discover effective therapeutics and preventive tools, including antiviral candidates and vaccines, to control this virus [12,13,14]. It is a great challenge for scientists to find antiviral agents and they are preferred to be natural products to have fewer side effects and be safer for different ages treated for this disease and improve immune response. Many medicinal plants provide a wealth of different chemicals, including antiviral activity [15,16,17,18]. Notably, the research on plant-based drugs is growing daily [19,20].

Three available medicinal plants well-known for their traditional medical use in Egypt were selected. They were *Artemisia judaica* L. (known as shih-Balady), *Azadirachta indica* (known as neem), and *Sophora tomentosa* (known as yellow necklacepod), and they have been known for harboring broad-spectrum antiviral, immune-stimulatory, and anti-inflammatory activities. These plant species are traditionally claimed to cure and/or reduce symptoms of various disorders including infectious diseases of animals and humans among folk cultures [21,22,23,24,25].

The selected antiviral agent must be safe and adequate, and the cost should be preferably low when used for prophylaxis and treatment of the virus. In our present study, the selected plant was *Artemisia judaica* (*A. judaica*) L. which has enjoyed a reputation among herb experts in Egypt (desert and coast) and is recommended as a healer plant in traditional medicine by Bedouins [26]. In Egypt, natural products have been frequently used in combination with conventional medicine to treat acute respiratory diseases. The plant is characterized by the presence of anti-inflammatory, antioxidant, antimicrobial, antihelicobacter, and broad-spectrum antiviral constituents. Shih-Balady has a history of being safe and easily available for therapies [27]. Previous work has highlighted the potential of *A. indica* as a candidate for plant-based prototypes that carry antiviral and immunogenic features against respiratory viral infections [27,28]. 

*Sophora tomentosa* (*S. tomentosa*) L. is an erect evergreen shrub; common names are yellow necklacepod, yellow *Sophora*, or Eve’s necklace. The overall natural range of *S. tomentosa* L. comprises just about every tropical and subtropical coast in the world. Many reports indicated that *S. tomentosa* extract contained polyphenolic compounds as the main phytochemicals. The main activities of these phytochemicals were to regulate immune function, suppress inflammation, and reduce lung injury by regulating multiple targets and signaling pathways [29]. Out of the tested 64 natural compounds, against SARS-CoV helicase, which plays an important role in the replication, transcription, and translation of viral genomes, the polyphenolic compounds were found to be the most promising members [30]. The chemical review on necklacepod reveals the presence of diverse bioactive compounds, mainly the isoprenylated flavonoids, isoflavonones, flavones, flavonols, and their glycosides. Such compounds have been reported to have diverse bioactivities including antioxidant and immunoregulatory [31]. Several studies have investigated the activity of phytochemicals against coronaviruses.

Different parts of the plants possess diverse compounds and the extraction of bioactive antiviral compounds is influenced by the solvent used. Ethyl acetate and dichloromethane were reported as the best solvents to be used to obtain an extract from many medicinal plants with potent antiviral activities [18,30]. The highest antiviral activity of several medicinal plants was associated with the crude ethyl acetate and/or dichloromethane extract, indicating the possibility of synergism among the antiviral constituents of the extract which may act by a different mode of action [32]. So, the ethyl acetate/dichloromethane (1:1, *v*/*v*) extract of each medicinal plant was used in the current study. 

There is no doubt that the in silico studies in the process of drug discovery constitute very important and crucial pathways for the rapid introduction of new drugs [33,34]. Computational methods help scientists to save effort and time [7,35,36]. Notably, molecular docking is one of the most widely used computational methods to predict and/or explain the mechanism of action for a certain ligand against a specific receptor [37,38,39].

**Figure 1:**
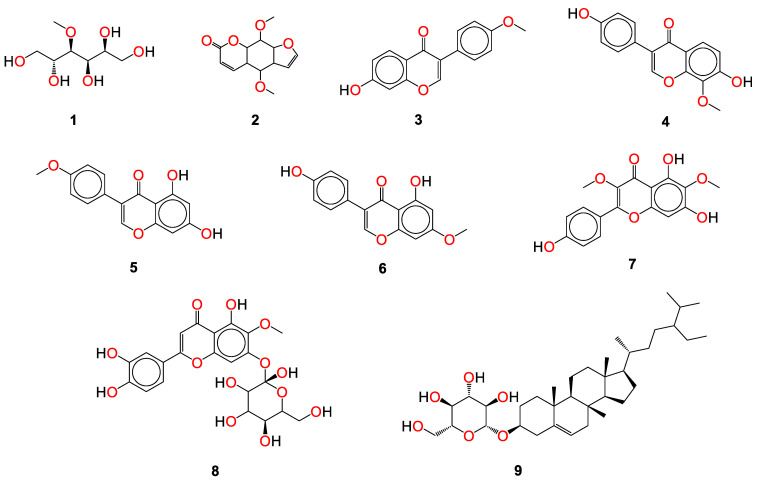
The isolated compounds from *S. tomentosa* L. leaves. 4-*O*-methyl sorbitol (**1**), 5,8-dimethoxypsoralen (**2**), formononetin (**3**), genistein 4’-methyl ether (**4**), 4’,5-dihydroxy-7-methoxygenistein (**5**), 8-methoxy daidzin (**6**), 5,7,4’-trihydroxy-3,6-dimethoxy flavone (**7**), 6-methoxy-7-*O*-β-D-glucoside apigenin (**8**), and daucosterol (**9**).

Herein, a comparative screening of the selected plants to evaluate their potential anticoronavirus activities against MERS-CoV propagation was carried out. Their cytotoxic activities were tested in Vero-E6 cells and the plot of % cytotoxicity versus sample concentration was used to calculate the concentration which exhibited 50% cytotoxicity (CC_50_). A plaque reduction assay was employed using the safe dose of each extract to evaluate its effect on virus propagation. Furthermore, the work was extended to study the possible mode of action of virus inhibition at three different levels: viral replication, viral adsorption, and virucidal activity with different safe concentrations. Additionally, isolation and structural identification of the compounds of *S. tomentosa* which were proposed to cause the antiviral activity against MERS and SARS were carried out. Finally, the anti-SARS-CoV-2 activities of the nine isolated and identified compounds from *S. tomentosa* (Figure 1) were recorded as well.

## 2. Results

### 2.1. Phytochemical Investigation 

The DCM/EA extract from the leaves of *S. tomentosa* was fractionated on a Si column, followed by consecutive purification steps on Si and Sephadex columns to yield one methyl derivative of sugar alcohol (**1**), one linear furanocoumarin (**2**), four isoflavonoids (**3**–**6**), two flavonoids (**7** and **8**), and one sterol glycoside **9** (Figure 1). The isolated compounds were identified as 4-*O*-methyl sorbitol (**1**), 5,8-dimethoxypsoralen (**2**), formononetin (**3**), genistein 4’-methyl ether (**4**), 4’,5-dihydroxy-7-methoxygenistein (**5**), 8-methoxy daidzin (**6**), 5,7,4’-trihydroxy-3,6-dimethoxy flavone (**7**), 6-methoxy-7-*O*-β-D-glucoside apigenin (**8**), and daucosterol (**9**). 

#### Identification of Isolated Compounds from *S. tomentosa*

In the present study, the promising extract of *S. tomentosa* leaves was subjected to a phytochemical investigation, which led to the isolation of nine compounds. The structure of each compound was determined by a variety of spectroscopic methods. A methyl derivative of sugar alcohol: 4-*O*-methyl sorbitol (1) [40], one linear furanocoumarin: 5,8-dimethoxypsoralen (**2**) [41,42], four isoflavonoids [43,44]: formononetin (**3**), genistein 4’-methyl ether (**4**), 4’,5-dihydroxy-7-methoxygenistein (**5**), 8-methoxy daidzin (**6**), two flavonoids: 5,7,4’-trihydroxy-3,6-dimethoxy flavone (**7**), 6-methoxy-7-*O*-β-D-glucoside apigenin (**8**), and one sterol glucoside [45,46]: daucosterol (**9**) were isolated and identified (Figure 1). Spectral data of the isolated compounds (**3**, **5**, and **9**) from *S. tomentosa* are depicted in the Supplementary Data (SI 1 and SI 2).

**Compound 1:** R_f_: 0.83 (S_1_); ^1^H-NMR (500 MHz, DMSO-d_6_): δ_ppm_ 3.66 (1H,t, H-3), 3.57 (1H, ddd, *J* = 3.6, 3.6, 6.2 Hz, H-2), 3.54 (1H, dd, *J* = 11.8, 3.8 Hz, H-6α), 3.51 (1H, dd, *J* = 12.3, 5.0 Hz, H-6β), 3.50 (1H, ddm, *J* = 5.0, 1.1Hz, H-5), 3.47 (3H,s, O-CH_3_-4), 3.46 (1H, dd, *J* = 12.3, 3.6 Hz, H-1α), 3.36 (1H, dd, *J* = 12.3, 3.6 Hz, H-1β), 3.02 (1H,t, H-4). ^13^C-NMR (125 MHz, DMSO-d_6_): δ_ppm_ 83.8 (C-4), 72.6 (C-2), 72.2 (C-3), 71.7 (C-5), 70.8 (C-1), 70.0 (C-6), 59.6 (O-CH_3_-4).

**Compound 2:** R_f_: 0.53, m.p. 148–51 °C, UV λ_max_ (nm): (MeOH): 246, 268, 310; EI–MS: *m*/*z* 246 [M^+^, C_13_H_10_O_5_], *m*/*z* 231 [M^+^–CH_3_], 203 and 175 [-CO], 160 [-CH_3_]; ^1^H–NMR (CDCl_3_, 300 MHz): δ8.12 (H–4, d, *J* = 9.5 Hz), δ7.62 (H–7, d, *J* = 2.5 Hz), δ7.05 (H–6, d, *J* = 2.5 Hz), δ6.35 (H–3, d, *J* = 9.5 Hz) and δ 4.13 (s, 5–OCH_3_), δ 4.09 (s, 8–OCH_3_).

**Compound 4:** R_f_: 0.91 (S_3_) and 0.32 (S_4_), m.p. 210–213 °C; UV λ_max_ (nm): (MeOH): 263 and 332 (sh), (+NaOMe): 272 and 330, (+AlCl_3_): 271, 312 (sh) and 376, (+AlCl_3_/HCl): 271, 312 (sh) and 371, (+NaOAc): 273 and 327, (+NaOAc/H_3_BO_3_): 263 and 332 (sh). ^1^H-NMR (400 MHz, DMSO-d_6_), δ_ppm_ 8.38 (1H, s, H-2), 7.52 (2H, d, *J* = 8.72 Hz, H-2’/6’), 7.03 (2H, d, *J* = 8.76 Hz, H-3’/5’), 6.22 (1H, d, *J* = 2.04 Hz, H-8), 6.41 (1H, d, *J* = 2.04 Hz, H-6), 3.77 (3H, s, O-CH_3_). ^13^C-NMR (100 MHz, DMSO-d_6_), δ_ppm_ 180.5 (C-4), 164.6 (C-7), 162.3 (C-5), 159.5 (C-4’), 158.3 (C-9), 154.6 (C-2), 130.5 (C-2’/ 6’), 123.2 (C-3), 122.5 (C-1’), 114.4 (C3’/5’), 104.6 (C-10), 99.6 (C-6), 94.4 (C-8), 55.8 (O-CH_3_).

**Compound 6:** R_f_: 0.63 (S_3_); 0.35 (S_4_), m.p 234–236 °C; UV λ_max_ (nm): (MeOH): 255 and 312 (sh), (+NaOMe): 257, 371 (sh) and 322, (+AlCl_3_): 259 and 305 (sh), (+AlCl_3_/HCl): 255, 304 (sh) and 363, (+NaOAc): 254 and 323, (+NaOAc/H_3_BO_3_): 252 and 317 (sh). ^1^H-NMR (500 MHz, DMSO-d_6_), δ_ppm_ Aglycone: 8.22 (1H, s, H-2), 7.46 (2H, d, *J* = 8.2 Hz, H-2’/6’), 6.98 (2H, d, *J* = 8.2Hz, H-3’/5’), 8.14 (1H, d, *J* = 9.2 Hz, H-6), 7.49 (1H, d, *J* = 9.2 Hz, H-5), 3.72 (3H, s, O-CH_3_). Sugar: 5.06 (1H, d, *J* = 7.6 Hz, H-1’’), 3.52–3.51 (m, rest of sugar protons). ^13^C-NMR (125 MHz, DMSO-d_6_), δ_ppm_ 175.3 (C-4), 161.7 (C-7), 159.7 (C-4’), 157.2 (C-9), 154.0 (C-2), 130.8 (C-2’/6’), 127.2 (C-5), 123.8 (C-3), 118.7 (C-1’), 116.4 (C-10), 114.3 (C-6), 103.7 (C-3’/5’), 100.6 (C-8), 100.2 (C-1’’), 77.9 (C-5’’), 76.8 (C-3’’), 73.9 (C-2’’), 70.2 (C-4’’), 61.3 (C-6’’), 55.9 (O-CH_3_).

**Compound 7:** R_f_: 0.49 (S_1_), 0.58 (S_5_), m.p. 211–214 °C; UV spectral data: λmax, nm (MeOH): 273, 340; (+NaOMe): 278, 326, 402; (+AlCl_3_): 276, 305 sh, 362, 407 sh; (+AlCl3/HCl): 282, 308 sh, 360, 405 sh; (+NaOAc): 271, 368; (+NaOAc+H_3_BO_3_): 271, 303 sh, 342; ^1^H-NMR (DMSO-d6, 300 MHz): δppm 12.76 (1H, s, OH-5), 7.93 (2H, d, *J* = 8.7 Hz, H-2’/6’), 6.91 (2H, d, *J* = 8.7 Hz, H-3’/5’), 6.54 (1H, s, H-8), 3.79 (s, 3H, OCH3-6), 3.76 (3H, s, OCH3-3); ^13^C-NMR (DMSO-d6, 75 MHz): δppm 178.2 (C-4), 160.3 (C-4’), 157.1 (C-7), 155.8 (C-2), 152.2 (C5), 151.7 (C-9), 137.0 (C-3), 131.0 (C-6), 130.4 (C-2’/6’), 120.5 (C1’), 115.7 (C-3’/5’), 104.6 (C-10), 93.8 (C-8), 59.7 (OCH3-6), 59.8 (OCH3-3).

**Compound 8:** R_f_: 0.54 (S_3_), 0.36 (S_4_); UV spectral data: λmax, nm, MeOH: 216, 278, 336; (+NaOMe): 237, 269, 392; (+NaOAc): 230, 274, 332, 398 (sh); (+NaOAc+H_3_BO_3_): 230, 274, 332, (+AlCl3): 229 (sh), 282, 302, 362; (+AlCl3-HCl): 228 (sh), 282, 302, 362. ^1^H-NMR (300 MHz, DMSO-d6) δppm 7.98 (2H, d, *J* = 8.4 Hz, H-2’/6’), 7.03 (1 H, s, H-3), 7.06 (2H, d, *J* = 8.4 Hz, H-3’/5’), 6.82 (1 H, s, H-8), 5.13 (1H, d, *J* = 7 Hz, H-1’’), 3.77 (3 H, s, OCH_3_), 3.9–3.4 (m, remaining sugar protons); ^13^C-NMR (75 MHz, DMSO-d_6_) δ ppm 182.7 (C-4), 165.4 (C-2), 162.2 (C-4’), 157.0 (C-7/ 9), 152.9 (C-5), 133.1 (C-6), 129.1 (C-2’/6’), 121.7 (C-1’), 116.6 (C-3’/5’), 106.4 (C-10), 103.1 (C-3), 101.3 (C-1’’), 95.4 (C-8), 77.7 (C-5’’), 77.2 (C-3’’), 73.6 (C-2’’), 70.2 (C4’’), 61.8 (C-6’’), 61.3 (OCH_3_).

It is worth mentioning that compounds **1**, **6,** and **8** were isolated for the first time from the *Sophora* genus. All compounds were isolated for the first time from this species except compounds **3** and **5**, while compound **9** was recently isolated from *S. mollis* (Royle) Graham Ex Baker [47].

### 2.2. Biological Activity Evaluations 

#### 2.2.1. Antiviral Activity for Three Medicinal Plants against MERS-CoV by Plaque Reduction Assay

**Figure 2:**
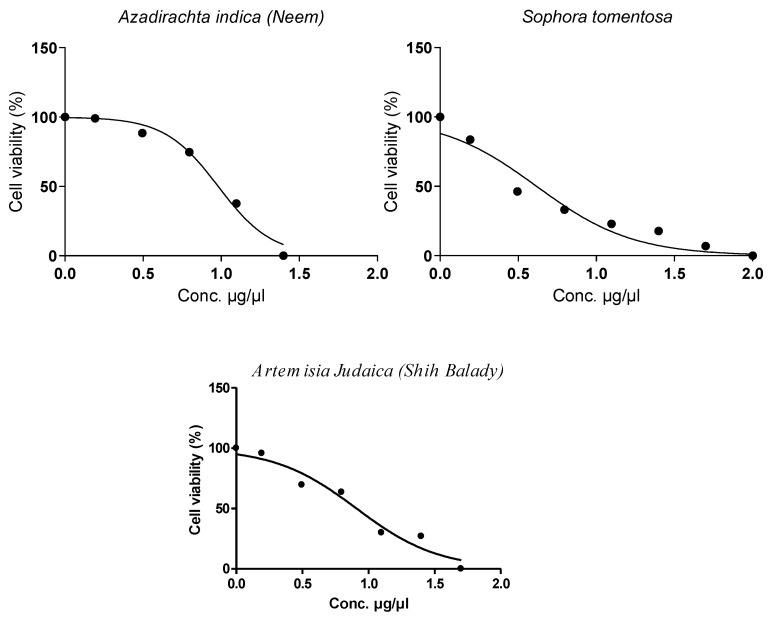
Cytotoxicity percentage and the concentration which exhibited 50% cytotoxic concentration (CC_50_) of *A. indica* (neem), *A. judaica* (shih-Balady), and *S. tomentosa* extracts by MTT assay which was used with CC_50_ of 22.52, 31.63, and 20.86 µg/mL, respectively.

In the present study, in searching for new anti-MERS-CoV agents, the study started with measuring the cytotoxic activity of each extract of the three selected plants in Vero-E6 cells using an MTT assay with some modification [48]. The concentration which exhibited 50% cytotoxic concentration (CC_50_) was calculated and was found to be equal to 22.52, 31.60, and 20.86 µg/mL for *A. indica* (Neem), *A. judaica*, and *S. tomentosa*, respectively, Figure 2. 

According to the results of the cytotoxicity assay (Figure 2) to determine CC_50_, different safe concentrations were selected to start plaque reduction assays for each extract against MERS-CoV virus propagation [49] (Table 1).

Table 1 represents the antiviral effects of the three plants’ extracts after being measured using a plaque reduction assay. The antiviral effects were from 96 to 88% for *Sophora* with concentrations of 12.50 to 3.13 µg/mL. On the other hand, they were from 92 to 85% for *Artemisia* with concentrations of 12.50 to 3.13 µg/µL. However, the lowest effect was for neem with 54% inhibition at the highest concentration (3.13 µg/mL).

Investigation of the chemical composition of the extract of *S. tomentosa* revealed the presence of four isoflavonoids (**3–6**). The isoflavonoids are an important polyphenolic subclass of flavonoids with a skeleton based on a 3-phenylchroman structure and their antiviral powers have been proven in several scientific reports before. Some isoflavonoids and flavonoids have been reported as potential candidates against viral infection [50]. The current results demonstrated that necklacepod plant extract showed 96% inhibition against the MERS-related coronavirus. This is in agreement with the previous results that showed that the flavones (luteolin, quercetin, and apigenin) isolated from *S. tomentosa* and *Artemisia judaica* L. were reported to inhibit severe acute RS-CoV 3CL^pr^° activity with IC_50_ values of 20.2, 23.8, and 280.8 μM, respectively [51]. Polyphenols (flavonoids) attack viral proteins present in the viral membrane or inside the virus particle. Phenolics are active against free viral particles but not—or to a lesser degree—after a virus has entered a host cell [52]. Limited data associated with the antiviral activities of neem may be due to its phytoconstituents not having a promising effect of as antivirals. 

In the present study, the highest inhibition percentage (96%) was recorded for *S. tomentosa* (necklacepod, of CC_50_ equal to 20.86 µg/µL), Figure 2.

#### 2.2.2. Mode of Action against MERS-CoV

**Figure 3:**
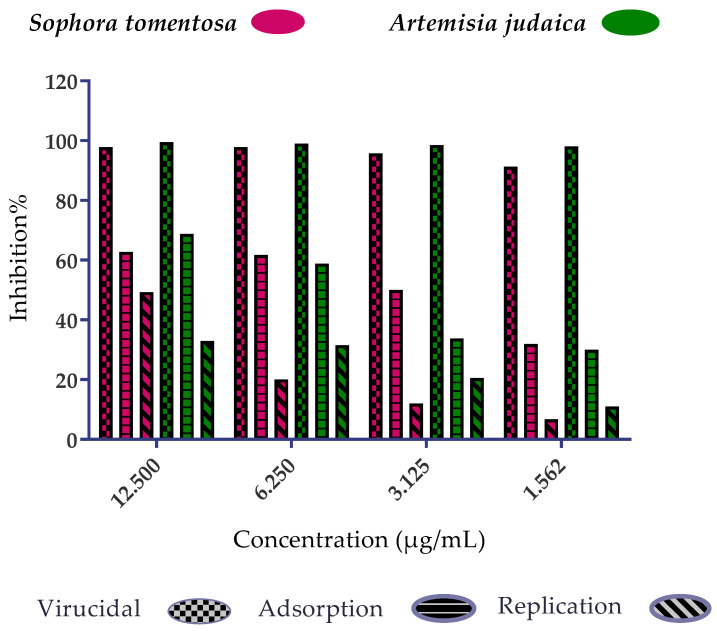
Mode of action for the two promising medicinal plants: *S. tomentosa* (Crude) and *A. judaica* (known shih-Balady), with different safe concentrations against MERS-CoV isolates (NRCE-HKU270 (Accession Number: KJ477103.2)). The virucidal effect was the main mode of action for the two extracts but has less effect on viral adsorption and a very low effect on viral replication.

The possible mode of action for herbal products may be investigated at three different levels: inhibition of viral replication, viral adsorption, and virucidal activity. Herein, the mode of action for the most promising medicinal plants with different safe concentrations was illustrated for both *S. tomentosa* and *A. judaica* (Figure 3). 

The results showed that they achieved 99.5% and 92% inhibition effects at 1.56 µg/mL for *S. tomentosa* and *A. judaica*, respectively, with virucidal as the main mode of action. This may be attributed to their direct effect on the virus which lost the ability of infectivity. So, the two plants have a virucidal effect more than the effect on viral adsorption to the cells and have less effect on viral replication.

#### 2.2.3. Comparison between the Antiviral Activity of *S. tomentosa* against MERS-CoV and SARS-CoV-2

The antiviral activity of *S. tomentosa* (necklacepod) was illustrated by plaque reduction assay against MERS-CoV isolate compared with SARS-CoV-2 isolate as depicted in Table 2. The results showed that antiviral activity against SARS-CoV-2 was very high (100%) and the extract succeeded in achieving full inhibition of viral propagation at different concentrations (12.50 and 6.25 µg/mL). On the other hand, it showed a slightly lower inhibition against MERS-CoV (96%) at the highest concentration (12.50 µg/mL).

#### 2.2.4. Antiviral Activity of the Isolated and Identified Compounds from *S. tomentosa* L. against SARS-CoV-2 by Crystal Violet Assay

**Figure 4:**
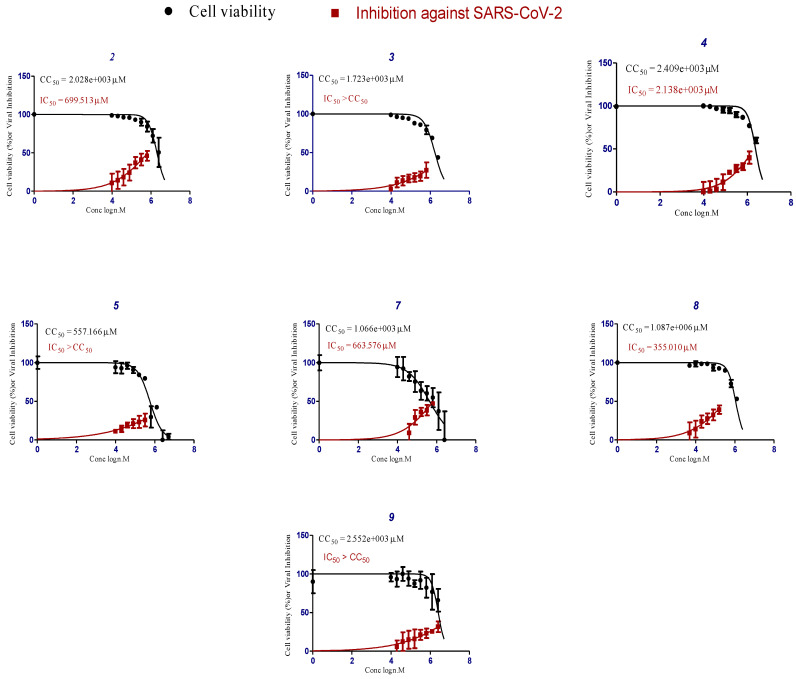
CC_50_ and IC_50_ of the isolated and identified compounds from *S. tomentosa* against SARS-CoV-2. 5,8-Dimethoxypsoralen (**2**), formononetin (**3**), genistein 4’-methyl ether (**4**), 4’,5-dihydroxy-7-methoxygenistein (**5**), 5,7,4’-trihydroxy-3,6-dimethoxy flavone (**7**), 6-methoxy-7-*O*-β-D-glucoside apigenin (**8**), and daucosterol (**9**).

The crystal violet assay to determine both the CC_50_ and IC_50_ of the isolated compounds from *S. tomentosa* L. against SARS-CoV-2 was applied (Figure 4). 

Generally, the highest anti-SARS-CoV-2 activity of *S. tomentosa* was associated with the crude ethanolic extract, indicating the possibility of synergism between the antiviral phytochemicals **1–9** of the extract. 

Herein, compound **4** (genistein 4’-methyl ether) was found to achieve superior anti-SARS-CoV-2 activity with an IC_50_ value of 2.13 µm. Moreover, compound **8** (6-methoxy-7-*O*-β-D-glucoside apigenin) showed a lower anti-SARS-CoV-2 activity with an IC_50_ value of 355.01 µm. However, both compounds [5,8-dimethoxypsoralen (**2**) and 5,7,4’-trihydroxy-3,6-dimethoxy flavone (**7**)] showed the lowest activities against SARS-CoV-2 with IC_50_ values of 699.51 and 663.57 µm, respectively. 

#### 2.2.5. Mode of Action of *S. tomentosa* L. against SARS-CoV-2

**Figure 5:**
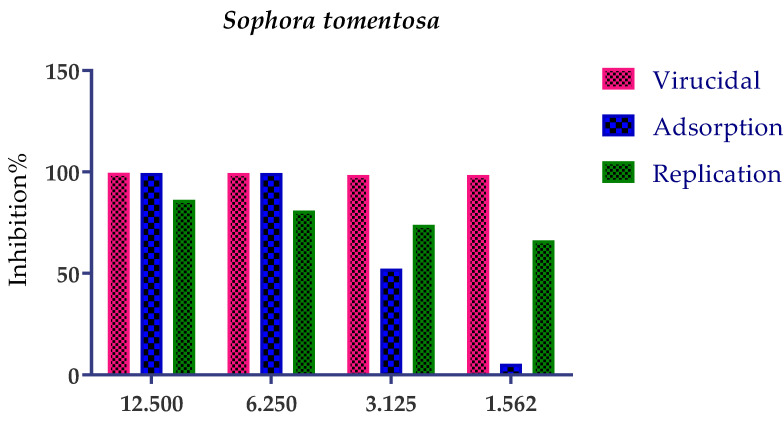
Mode of action for the most promising medicinal plant *S. tomentosa* (crude) with different safe concentrations against SARS-CoV-2 isolate (*h*CoV-19/Egypt/NRC-3/2020). *S. tomentosa* had a promising effect (>99%) against SARS-CoV-2 by two mechanisms of action (virucidal and adsorption) with 12.50 and 6.25 µg/mL. On the other hand, its efficacy by adsorption decreased when decreasing the concentration but was still high (>99%) with a virucidal mechanism at 3.125 and 1.562 µg/mL.

It was important to test the mode of action of *S. tomentosa* against the SARS-CoV-2 isolate (Figure 5). The results showed that two mechanisms of action (virucidal and adsorption) were effective at 12.50 and 6.25 µg/mL with an inhibition percent of more than 99%. On the other hand, the extract’s efficacy by adsorption decreased when decreasing the concentration but was still high with a virucidal mechanism of action (>99%) at 3.12 and 1.56 µg/mL.

#### 2.2.6. Antiviral Activities for Crude *S. tomentosa* L. against Both SARS-CoV-2 and MERS-CoV by Crystal Violet Assay 

**Figure 6:**
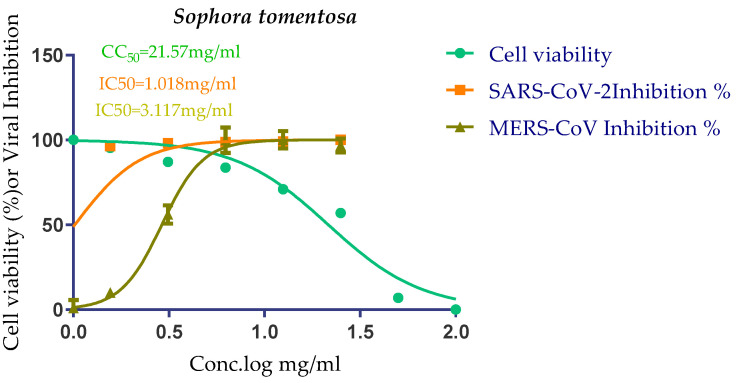
CC_50_ and IC_50_ antiviral activity of *S. tomentosa* against SARS-CoV-2 and MERS-CoV. The IC_50_ of each test was calculated using nonlinear regression analysis in triplicate for each concentration used. The best-fitting line was drawn between log concentrations and viral inhibition % using GraphPad Prism software.

Both the 50% cytotoxic concentration (CC_50_) and the 50% inhibitory concentration (IC_50_) were measured in the same conditions for *S. tomentosa* (Figure 6). 

The CC_50_ of *S. tomentosa* was recorded to be 21.57 µg/mL. Furthermore, the IC_50_ values against SARS-CoV-2 and MERS-CoV were found to be 1.01 and 3.11 µg/mL, respectively. The therapeutic indexes for *S. tomentosa* against SARS-CoV-2 and MERS-CoV were 21.18 and 6.92, respectively, as well. 

Based on the above, we can conclude that *S. tomentosa* is more active against SARS-CoV-2 and it may be considered a promising anti-SARS-CoV-2 therapy after more advanced preclinical and clinical studies.

### 2.3. Docking Studies

The X-ray structures of the S and M^pr^° receptors of SARS-CoV-2 were visualized and studied carefully based on the data introduced in the PDB and literature. It was clear that Asp80 is one of the most crucial amino acids in the S binding pocket of SARS-CoV-2 [53]. However, Glu166 is the most crucial amino acid for the inhibition of the dimeric M^pr^° pocket of SARS-CoV-2 [54].

Herein, the two most biologically active isolates (**4** and **8**) were selected for a further deep investigation to propose their expected mechanism of action and try to explain their inhibitory activity as well. Their scores, RMSD, 3D binding interactions, and 3D positioning inside the binding pockets of the S and M^pr^° pockets of SARS-CoV-2, besides the co-crystallized O6K inhibitor of M^pr^°, are represented in Table 3. 

Regarding the docking process within the S binding pocket of SARS-CoV-2, we can observe that:

(a) Compound **4** was stabilized inside the S binding pocket through the formation of one H-bond with crucial amino acid Asp80 and with a binding score of −5.71 kcal/mol. This indicates the large binding affinity of the mentioned compound which does not need more binding sites and has an expected superior intrinsic activity as well.
(b) On the other hand, compound **8** showed the formation of two H-bonds with Asp80 and Asn137 amino acids. Its binding score was found to be −7.03 kcal/mol.

However, concerning the docking process towards the M^pr^° receptor of SARS-CoV-2, we can show that:

(a) The docked O6K inhibitor of the dimeric M^pr^° binding pocket formed three H-bonds with Glu166, Asn142, and Ser1 amino acids. Moreover, it achieved a score of −8.98 kcal/mol.
(b) Notably, compound **4** bound the crucial Glu166 amino acid with one H-bond which was enough to stabilize itself and produce its inhibitory effect with a binding score of −6.44 kcal/mol.
(c) Furthermore, compound **8** formed three H-bonds with Glu166, Asn142, and Gln192 with a binding score of −7.36 kcal/mol.

Based on the above, we can conclude that the most biologically active compounds (**4** and **8**) formed H-bonds with the crucial amino acids which are important for the inhibition of the S and M^pr^° receptors of SARS-CoV-2 (Asp80 and Glu166, respectively). This greatly recommends the proposed mechanisms of action for the studied isolates as SARS-CoV-2 inhibitors targeting both the S and M^pr^° receptors. Notably, the docking results showed near matching with the previously discussed in vitro results as well.

## 3. Materials and Methods

### 3.1. Plant Materials

The pubescent leaves of shih-Balady (*Artemisia judaica* L., family Asteraceae) were purchased from an Egyptian market. A collection of the leaves of neem (*Azadirachta indica* A. juss., family: Meliaceae) was made at the Ministry of Agriculture, Giza, Egypt. The necklacepod (*Sophora tomentosa* L., family: Fabaceae) was collected from El Qanater, Qalyubia Governorate. Authentication was performed by Treas Labib, consultant of plant taxonomy at the Ministry of Agriculture and ex-director of El-Orman Garden (Giza, Egypt), and by Sherif S. El-Khanagry, Department of Flora and Phytotaxonomy Research Unit of the Agricultural Museum, Ministry of Agriculture of Giza, Egypt. Authentic reference material was available at the Department of Chemistry of Natural Compounds.

### 3.2. Preparation of Extracts for Antiviral Assays

The fresh leaves of each plant were dried in the shade in an air draft at room temperature. Each plant material (100 g) was separately refluxed in a dichloromethane-ethyl acetate (DCM/EA, 1:1, *v*/*v*) mixture (three extractions, each for 8 h with 1.25 L). The collected solution was filtered and dried in a vacuum to yield brown, greenish-brown, and dark brown amorphous residue of the DCM/EA extract of *A. judaica* (DCM/EA-Ar), *A. indica* (DCM/EA-Az), and *S. tomentosa* (DCM/EA-S), respectively. The percentage yields were 28.6, 34.2, and 11.6%, respectively. Part of each extract was re-dissolved in dimethyl sulfoxide (DMSO; Sigma-Aldrich, Merck KGaA, Darmstadt, Germany), and the stocks (100 µg/mL) were stored at −20 °C until subsequent use.

### 3.3. Phytochemical Study

#### 3.3.1. General

The NMR spectra were recorded at 300, 400, 500 *(*^1^H), and *75,* 100, 125 (^13^C) MHz on a Varian Mercury 300, Bruker High-Performance Digital FT-NMR 400 Avance III, and JOEl ECA 500 MHz spectrometer, respectively, using a convenient solvent. The chemical shifts (δ) are reported in parts per million (ppm) and coupling constants (*J*) in Hz. Herein, Gallenkamp electrothermal melting point apparatus and electrothermal digital apparatus were used. EI-MS spectra were taken on HP; MS-5988. The UV analyses of the pure samples were recorded, separately, as MeOH solutions and with different diagnostic UV shift reagents on a Shimadzu UV 240 (P/N 240-5800) UV–visible spectrophotometer.

#### 3.3.2. Material for Chromatography

Column chromatography (CC) was performed using silica gel (Si) 60 mesh of 35–60 and 0.063–0.200 mm (E. Merck, Darmstadt, Germany) and Sephadex LH-20 (Pharmacia, Uppsala, Sweden). Precoated silica gel 60 F254 plates (Merck) for thin-layer chromatography (TLC) were used. TLC spots were visualized under UV (254 nm) and sprayed with a convenient spray reagent. For paper chromatography, Whatman No. 1 paper sheets (Whatman Ltd., Maidstone, Kent, UK) were used.

#### 3.3.3. Chemicals

The chemicals were high analytical grade products from Sigma (St. Louis, MA, USA), Merck (Darmstadt, Germany), BDH (Poole Dorset BH15 1TD, UK), and Fluka (Fluka Biochemika, Buchs, Switzerland). 

#### 3.3.4. Solvent Systems and Spray Reagents 

S_1_: EtOAc/*HCOOH*/CH_3_COOH/H_2_O (100:11:11:27, *v*/*v*/*v*/*v*), S_2_: petroleum ether/MeOH; S_3_: [n-BuOH-HOAc-H2O (4:1:5, *v*/*v*/*v*, top layer)], S_4_: 15% aqueous HOAc, S_5_: benzene/EtOAc (6:4 *v*/*v*), and S_6_: CH_2_Cl_2_-MeOH (8.5:1.5 *v*/*v*) were used for TLC. All solvents used for extractions and chromatographic separations were of analytical grade. The visualization of spots was performed by spraying with the spray reagents, R_1_: iodine/potassium iodide (I_2_/KI) and R_2_: anisaldehyde–sulfuric acid (0.5 mL *p*-anisaldehyde, 85 mL methanol, 10 mL glacial acetic acid, and 5 mL concentrated sulfuric acid was added cautiously), R_3_: AlCl_3_ spray reagent (1 g powder of AlCl_3_ in 100 mL of ethanol).

#### 3.3.5. Extraction and Isolation 

The procedure depends on the isolation of compounds from DCM/EA (1:1, *v*/*v*) after the ethanol precipitation process for purifying sugar substances from the crude aqueous alcohol extract. Briefly, the air-dried and powdered leaves of *S. tomentosa* (1900 g) were exhaustively extracted with ethanol (80%). The ethanolic extract was evaporated to dryness under reduced pressure to afford a greenish-gray gummy residue. The residue was treated with the addition of excess ethanol. A yellowish-white precipitate was produced (F. I). The precipitate was purified using Sephadex LH- 20 (MeOH as eluent) to give compound **1**. Compound **1** was obtained as white crystals (29 mg) soluble in water and is observed as a colorless spot in visible light and a purple spot under UV light. The remaining solution, once the precipitate has been filtered out, is known as the filtrate and was dried in vacuo to give 89.3 g. The dried filtrate was fractionated with DCM/EA (1:1, *v*/*v*). A part of the total DCM/EA-S (40 g) was column chromatographed on a Si column and was eluted with n-hexane containing an increasing amount of ethyl acetate (100:0 → 0:100). A number of fractions (Fr. II–Fr. VI) were afforded which were combined based on TLC monitoring. Compound **2** was obtained from Fr. II eluted with *n*-hexane/ethyl acetate (7.5:2.5) and it was purified by crystallization (methanol as eluent) and obtained as a white powder (19 mg). Fr. III was re-chromatographed on the Si column by elution with EtOAc: MeOH (10:0–7:3) and divided into five subfractions (Fr. 02-Fr. 06). Fr. 02 and Fr. 03 were, separately, subjected to repeated CC on Si with *n*-hexane/acetone to give two semi-pure compounds. Each compound was crystallized with methanol to give compounds **3** and **4** as a yellow amorphous powder (24 mg) and yellowish-white crystals (28 mg), respectively. Compound **6** (20 mg) was obtained from Fr. 04 after final purification was achieved through Sephadex LH-20 (methanol as eluent) as a yellow amorphous powder (14 mg). The subfraction Fr. 05 was subjected to preparative TLC on Si CC with CHCl_3_-Me_2_CO (8:1) to give compounds **5** and **7** as yellow amorphous powder (22 mg) and light-yellow crystals (16 mg), respectively. On the other hand, fractions Fr. IV and Fr. V were combined and subjected to Si CC eluting with a solvent system of CHCl_3_-MeOH-H_2_O (9:1:0.1) to give compound **8** (15 mg). Fr. VI led to the isolation of compound **9**. This fraction was subjected to repeat CC on Si with CHCl_3_/EtOAc to give a white crystalline solid (33 mg) of **9**. 

The purity of compounds **1**–**9** was checked by TLC using convenient solvent systems S_1_–S_6_. Spray reagents R_1_ and R_2_ were used for compounds **2** and **9**, respectively. R_3_ was used for compounds **3**–**8**. All compounds were characterized mainly by spectroscopic methods, UV, ^1^H, and ^13^C NMR, and a comparison of the melting points with authentic samples or those in the literature was carried out.

### 3.4. Virus and Cells

The cell type used in the study was Vero-E6 cells from the National Research Centre (NRC). The MERS-CoV isolates (NRCE-HKU270 (Accession Number: KJ477103.2)) were a virus that is infecting humans. Moreover, SARS-CoV-2 isolates (*h*CoV-19/Egypt/NRC-03/2020 (Accession Number on GSAID: EPI_ISL_430820)) were used. The isolates were approved by the ethics committee of the NRC (Giza, Egypt).

### 3.5. Biological Activity Evaluations

#### 3.5.1. Determination Titers of Viruses by Plaque Titration Assay

Plaque titration assay [55] was used for the determination of titers of MERS-CoV and SARS-CoV-2 to be used in other assays as the mode of action and plaque reduction assay. The full methodology is depicted in the Supplementary Material (SI 3).

#### 3.5.2. MTT Cytotoxicity Assay (CC_50_)

The cytotoxic activity of the extracts was tested in Vero-E6 cells by using the MTT method with minor modification [56]. The applied full methodology is described in the Supplementary Material (SI 4).

#### 3.5.3. Plaque Reduction Assay 

The assay was performed according to the Hayden et al. method as previously described [57]. The full methodology is represented in the Supplementary Material (SI 5).

#### 3.5.4. Mode of Action of Virus Inhibition

The possible mode of action of virus inhibition by the selected plants’ extracts was examined at three different stages of the virus propagation cycle and based on three main possible modes of action:

(i) Inhibition of budding and viral replication.
(ii) The ability of each extract to inhibit the attachment of the virus to infected cells—membrane fusion is known to block the viral entry (viral adsorption).
(iii) The direct effect of each extract is to inactivate the virus viability (virucidal activity).

Additionally, the above-mentioned modes of action could account for the recorded antiviral activities either independently or in combinations. In this regard, the interaction between the selected plants’ extracts and MERS-CoV could be explained through the following three different modes of action (Supplementary Material, SI 6):

##### Viral Replication

The viral replication assay was applied according to Kuo et al. [58] as described in the Supplementary Material (SI 6.1). 

##### Viral Adsorption

The viral adsorption assay using the Zhang et al. method [59] was performed as represented in the Supplementary Material (SI 6.2). 

##### Virucidal

The virucidal assay was carried out [60] as depicted in the Supplementary Material (SI 6.3). 

#### 3.5.5. Inhibitory Concentration 50 (IC_50_) Calculation

The inhibitory concentration 50 (IC_50_) of the examined extracts was tested in Vero-E6 cells according to the reported methodology [61] described in detail in the Supplementary Material (SI 7).

### 3.6. Docking Studies

The nine isolated compounds from *S. tomentosa* (**1**–**9**) were inserted in two separate docking processes against both the S and M^pr^° receptors of SARS-CoV-2 using the MOE 2019.012 suite [62,63]. This was carried out to propose their expected mechanism of action as anti-SARS-CoV-2 agents targeting the S and/or M^pr^° receptors. Additionally, the co-crystallized inhibitor of the M^pr^° receptor pocket (O6K, 10) was used in the M^pr^° docking process as a reference standard. 

#### 3.6.1. Validation of the MOE Program

**Figure 7:**
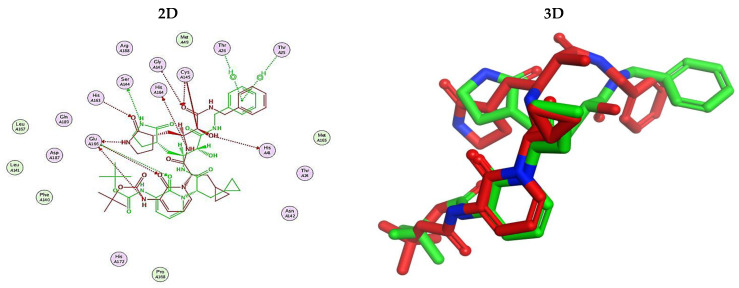
Superimposition of the redocked O6K inhibitor (green) over its native one (red).

This was carried out to confirm the validity of the docking program to be able to consider the presented docking results [64,65]. Therefore, the co-crystallized inhibitor of the M^pr^° receptor (O6K) was redocked within its binding pocket, and both its binding mode and root mean square deviation (RMSD) were studied. The MOE validation was concluded based on obtaining approximately the same binding mode of the redocked O6K (green) compared to its native one (red) as depicted in Figure 7, and the low value of RMSD (1.41).

#### 3.6.2. Preparation of the *S. tomentosa* Isolated Compounds 

The nine isolated compounds (**1–9**) from *S. tomentosa* were sketched using the ChemDraw program. Each compound was introduced individually into the MOE program window and prepared for docking as discussed before [66,67]. Then, the nine prepared isolates (**1–9**) were imported into two different databases in order to perform two separate docking processes. 

#### 3.6.3. Preparation of the S and M^pr^° Receptors of SARS-CoV-2

The X-ray structures of both the S and M^pr^° receptors of SARS-CoV-2 (IDs: 7FCD [53] and 6Y2G [54], respectively) were downloaded from the Protein Data Bank (PDB). Each protein was prepared as described earlier in detail [68,69]. 

#### 3.6.4. Docking of Each Database into the Corresponding Binding Pocket of SARS-CoV-2 

Each database was uploaded in a separate general docking process according to the previously discussed methodology [70,71]. Moreover, the best pose for each tested compound was selected according to the binding mode, score, and RMSD as well [72,73]. 

## 4. Conclusions

Three selected medicinal plants (*A. indica* (neem), *A. judaica*, and *S. tomentosa*) were screened against MERS-CoV using a plaque reduction assay. The highest viral inhibition percentage (96%) was recorded for *S. tomentosa* (known as yellow necklacepod) with CC_50_ of 20.86 µg/mL. Then, the mode of action for both *S. tomentosa* and *A. judaica* showed that they achieved 99.5% and 92% inhibition effects, respectively, at a concentration of 1.56 µg/mL, with virucidal as the main mode of action. Moreover, the antiviral activity of *S. tomentosa* against both MERS-CoV and SARS-CoV-2 using a plaque reduction assay was measured. It showed that the antiviral activity of *S. tomentosa* against SARS-CoV-2 was very high (100%) and the extract succeeded in achieving full inhibition for viral propagation at different concentrations (12.50 and 6.25 µg/mL). In addition, it showed a slightly lower inhibition against MERS-CoV (96%) at the highest concentration (12.50 µg/mL). Furthermore, the phytochemical investigation of the very promising *S. tomentosa* L. led to the isolation and structure determination of nine compounds (**1–9**) using different techniques. Notably, compound **4** (genistein 4’-methyl ether) was found to achieve superior anti-SARS-CoV-2 activity among other isolates with an IC_50_ value of 2.13 µm. Interestingly, it was important to test the mode of action of *S. tomentosa* against SARS-CoV-2. The results showed that two mechanisms of action (virucidal and adsorption) were effective at 12.50 and 6.25 µg/mL with an inhibition of more than 99%. On the other hand, the CC_50_ of *S. tomentosa* was recorded to be 21.57 µg/mL. Additionally, the IC_50_ values against SARS-CoV-2 and MERS-CoV were found to be 1.01 and 3.11 µg/mL, respectively. The therapeutic indexes for *S. tomentosa* against SARS-CoV-2 and MERS-CoV were 21.18 and 6.92, respectively, as well. Obviously, we can conclude that *S. tomentosa* is more active against SARS-CoV-2 and it may be considered a promising anti-SARS-CoV-2 therapy after more advanced preclinical and clinical studies. Finally, molecular docking studies clarified that the most biologically active compounds (**4** and **8**) showed the formation of H-bonds with the crucial amino acids which are important for the inhibition of the S and M^pr^° receptors of SARS-CoV-2 (Asp80 and Glu166, respectively). This greatly recommends the proposed mechanisms of action for the studied isolates as SARS-CoV-2 inhibitors targeting both the S and M^pr^° receptors.

## Figures and Tables

**Table 1:** Antiviral activity of three medicinal plants using plaque reduction assay against the MERS-CoV isolates (NRCE-HKU270 (Accession Number: KJ477103.2)).

Name of Plant	Conc. (µg/mL)	Viral Count (PFU/mL)	Viral Count after Treatment (PFU/mL)	Inhibition %
*Azadirachta indica* (Neem)	3.13	2.6 × 10^−5^	1.2 × 10^−5^	54%
	1.56		2.0 × 10^−5^	42%
	0.78		1.5 × 10^−5^	23%
*Artemisia judaica* (Shih-Balady)	12.50	2.6 × 10^−5^	2 × 10^−4^	92%
	6.25		3 × 10^−4^	88%
	3.13		4 × 10^−4^	85%
*Sophora tomentosa* (Yellow Necklacepod)	12.50	2.6 × 10^−5^	1 × 10^−4^	96%
	6.25		2 × 10^−4^	92%
	3.13		3 × 10^−4^	88%

PFU: Plaque-Forming Unit.

**Table 2:** Comparison between the antiviral activity of *S. tomentosa* (necklacepod) using plaque reduction assay against MERS-CoV isolate (NRCE-HKU270 (Accession Number: KJ477103.2)) and SARS-CoV-2 isolate (*h*CoV-19/Egypt/NRC-3/2020).

Conc (µg/mL)	MERS-CoV			SARS-CoV-2		
	Viral Count (PFU/mL)	Viral Count after Treatment (PFU/mL)	Inhibition %	Viral Count (PFU/mL)	Viral Count after Treatment (PFU/mL)	Inhibition %
12.50	2.6 × 10^5^	1 × 10^4^	96%	80 × 10^4^	0	100%
6.25		2 × 10^4^	92%		0	100%
3.12		3 × 10^4^	88%		1 × 10^4^	98.75%

**Table 3:** Binding scores, RMSD, 2D binding, and 3D positioning of compounds from *S. tomentosa* (**4** and **8**) inside the S and M^pr^° pockets of SARS-CoV-2, besides the co-crystallized O6K inhibitor of M^pr^°.

No.	Comp.	Receptor	^a^ S	RMSD	2D Interaction	3D Interaction	3D Positioning
4	Genistein 4’-methyl ether	S	−5.71	1.77	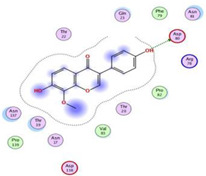	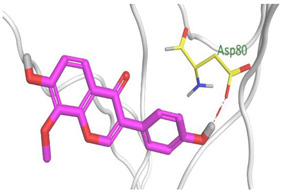	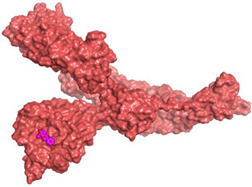
		M^pr^°	−6.44	0.80	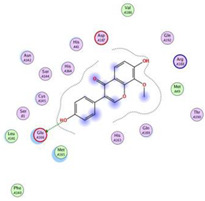	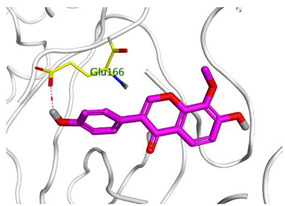	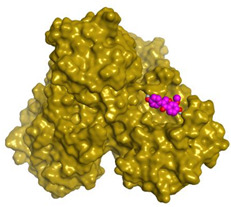
8	6-Methoxy-7-O-β-D-glucoside apigenin	S	−7.03	2.13	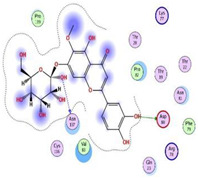	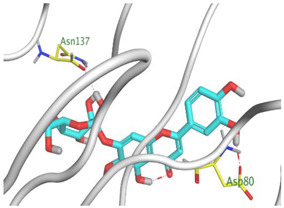	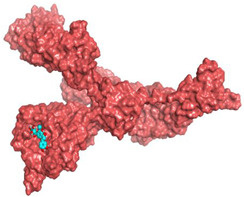
		M^pr^°	−7.36	1.12	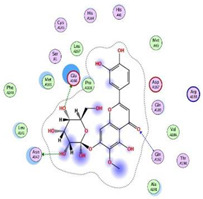	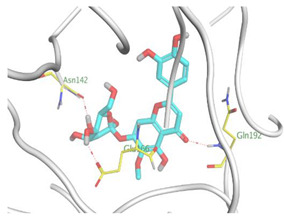	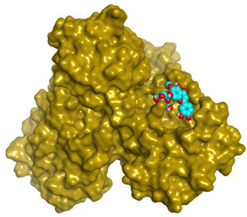
10	O6K	M^pr^°	−8.98	1.99	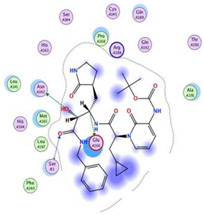	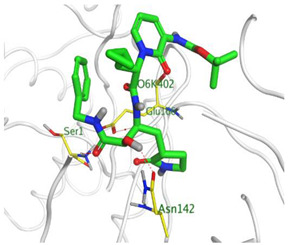	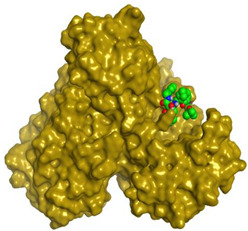

^a^ S: Score of a compound within the protein binding pocket (Kcal/mol).

## Data Availability

Not applicable.

## Supplementary Materials

The following supporting information can be downloaded at: https://www.mdpi.com/article/10.3390/metabo12111109/s1, SI 1. Identification of isolated compounds from *S. tomentosa*; SI 2. Spectral data of the isolated compounds from *S. tomentosa*; SI 3. Determination titers of viruses by plaque titration assay; SI 4. MTT cytotoxicity assay (CC_50_); SI 5. Plaque reduction assay; SI 6. Mode of action of virus inhibition (SI 6.1, SI 6.2, and SI 6.3); SI 7. Inhibitory concentration 50 (IC_50_) determination. References [12,13,14,16,33,35,56,57,58,59,60,61,74,75] are cited in the supplementary materials.

## References

[1] Al-Karmalawy A.A., Soltane R., Abo Elmaaty A., Tantawy M.A., Antar S.A., Yahya G., Chrouda A., Pashameah R.A., Mustafa M., Abu Mraheil M. (2021). Coronavirus Disease (COVID-19) Control between Drug Repurposing and Vaccination: A Comprehensive Overview. Vaccines.

[2] Ashour N.A., Elmaaty A.A., Sarhan A.A., Elkaeed E.B., Moussa A.M., Erfan I.A., Al-Karmalawy A.A. (2022). A Systematic Review of the Global Intervention for SARS-CoV-2 Combating: From Drugs Repurposing to Molnupiravir Approval. Drug Des. Dev. Ther..

[3] Rabaan A.A., Al-Ahmed S.H., Haque S., Sah R., Tiwari R., Malik Y.S., Dhama K., Yatoo M.I., Bonilla-Aldana D.K., Rodriguez-Morales A.J. (2020). SARS-CoV-2, SARS-CoV, and MERS-COV: A comparative overview. Infez Med.

[4] Roshdy W.H., Khalifa M.K., San J.E., Tegally H., Wilkinson E., Showky S., Martin D.P., Moir M., Naguib A., Elguindy N. (2022). SARS-CoV-2 Genetic Diversity and Lineage Dynamics in Egypt during the First 18 Months of the Pandemic. Viruses.

[5] Sarhan A.A., Ashour N.A., Al-Karmalawy A.A. (2021). The journey of antimalarial drugs against SARS-CoV-2: Review article. Inform. Med. Unlocked.

[6] Park S.E. (2020). Epidemiology, virology, and clinical features of severe acute respiratory syndrome-coronavirus-2 (SARS-CoV-2; Coronavirus Disease-19). Clin. Exp. Pediatr..

[7] Abdallah A.E., Alesawy M.S., Eissa S.I., El-Fakharany E.M., Kalaba M.H., Sharaf M.H., Abo Shama N.M., Mahmoud S.H., Mostafa A., Al-Karmalawy A.A. (2021). Design and synthesis of new 4-(2-nitrophenoxy)benzamide derivatives as potential antiviral agents: Molecular modeling and in vitro antiviral screening. New J. Chem..

[8] Abo Elmaaty A., Hamed M.I.A., Ismail M.I.B., Elkaeed E.S., Abulkhair H., Khattab M., Al-Karmalawy A.A. (2021). Computational Insights on the Potential of Some NSAIDs for Treating COVID-19: Priority Set and Lead Optimization. Molecules.

[9] Nascimento Junior J.A.C., Santos A.M., Quintans-Junior L.J., Walker C.I.B., Borges L.P., Serafini M.R. (2020). SARS, MERS and SARS-CoV-2 (COVID-19) treatment: A patent review. Expert Opin. Ther. Pat..

[10] Al-Karmalawy A.A., Alnajjar R., Dahab M., Metwaly A., Eissa I. (2021). Molecular docking and dynamics simulations reveal the potential of anti-HCV drugs to inhibit COVID-19 main protease. Pharm. Sci..

[11] Al-Karmalawy A.A., Dahab M.A., Metwaly A.M., Elhady S.S., Elkaeed E.B., Eissa I.H., Darwish K.M. (2021). Molecular Docking and Dynamics Simulation Revealed the Potential Inhibitory Activity of ACEIs Against SARS-CoV-2 Targeting the h ACE2 Receptor. Front. Chem..

[12] Elmaaty A.A., Eldehna W.M., Khattab M., Kutkat O., Alnajjar R., El-Taweel A.N., Al-Rashood S.T., Abourehab M.A.S., Binjubair F.A., Saleh M.A. (2022). Anticoagulants as Potential SARS-CoV-2 Mpro Inhibitors for COVID-19 Patients: In Vitro, Molecular Docking, Molecular Dynamics, DFT, and SAR Studies. Int. J. Mol. Sci..

[13] Elagawany M., Elmaaty A.A., Mostafa A., Abo Shama N.M., Santali E.Y., Elgendy B., Al-Karmalawy A.A. (2022). Ligand-based design, synthesis, computational insights, and in vitro studies of novel N-(5-Nitrothiazol-2-yl)-carboxamido derivatives as potent inhibitors of SARS-CoV-2 main protease. J. Enzym. Inhib. Med. Chem..

[14] Kutkat O., Moatasim Y., Al-Karmalawy A.A., Abulkhair H.S., Gomaa M.R., El-Taweel A.N., Abo Shama N.M., GabAllah M., Mahmoud D.B., Kayali G. (2022). Robust antiviral activity of commonly prescribed antidepressants against emerging coronaviruses: In vitro and in silico drug repurposing studies. Sci. Rep..

[15] El Gizawy H.A., Boshra S.A., Mostafa A., Mahmoud S.H., Ismail M.I., Alsfouk A.A., Taher A.T., Al-Karmalawy A.A. (2021). *Pimenta dioica* (L.) Merr. Bioactive Constituents Exert Anti-SARS-CoV-2 and Anti-Inflammatory Activities: Molecular Docking and Dynamics, In Vitro, and In Vivo Studies. Molecules.

[16] Soltane R., Chrouda A., Mostafa A., Al-Karmalawy A.A., Chouaïb K., dhahri A., Pashameah R.A., Alasiri A., Kutkat O., Shehata M. (2021). Strong Inhibitory Activity and Action Modes of Synthetic Maslinic Acid Derivative on Highly Pathogenic Coronaviruses: COVID-19 Drug Candidate. Pathogens.

[17] Abd-Alla H.I., Sweelam H.-t.M., Mohamed T.A., Gabr M.M., El-Safty M.M., Hegazy M.-E.F. (2017). Efficacy of extracts and iridoid glucosides from Pentas lanceolata on humoral and cell-mediated immune response of viral vaccine. Med. Chem. Res..

[18] Abd-Alla H.I., Abu-Gabal N.S., Hassan A.Z., El-Safty M.M., Shalaby N.M. (2012). Antiviral activity of Aloe hijazensis against some haemagglutinating viruses infection and its phytoconstituents. Arch. Pharmacal Res..

[19] Al-Karmalawy A.A., Farid M.M., Mostafa A., Ragheb A.Y.H., Mahmoud S., Shehata M., Shama N.M.A., GabAllah  M., Mostafa-Hedeab G., Marzouk M.M. (2021). Naturally Available Flavonoid Aglycones as Potential Antiviral Drug Candidates against SARS-CoV-2. Molecules.

[20] Zaki A.A., Al-Karmalawy A.A., El-Amier Y.A., Ashour A. (2020). Molecular docking reveals the potential of Cleome amblyocarpa isolated compounds to inhibit COVID-19 virus main protease. New J. Chem..

[21] Arbab A.H., Parvez M.K., Al-Dosari M.S., Al-Rehaily A.J. (2017). In vitro evaluation of novel antiviral activities of 60 medicinal plants extracts against hepatitis B virus. Exp. Ther. Med..

[22] Cong Y., Gross R., Zhou H., Frieman M., Bollinger L., Wada J., Hensley L.E., Jahrling P.B., Dyall J., Holbrook M.R. (2018). MERS-CoV pathogenesis and antiviral efficacy of licensed drugs in human monocyte-derived antigen-presenting cells. PloS ONE.

[23] Aly H.F., Abd-Alla H.I., Ali S.A., Aba-Alez R., Abu-Krisha M., Mamdouh M.M. (2017). Bioinformatics: Inflammatory cytokines and attenuation of diabetes hypercholesterolemia-induced renal injury using morning glory and necklace pod extracts. Asian J. Pharm. Clin. Res..

[24] Abd-Alla H.I., Heba-tollah M.S., El-Kashak W.A., El-Safty M.M. (2019). Evaluation of immune boosting properties and combating of multiple respiratory viral infections by fifteen Euphorbiaceae plant extracts. Pharmacogn. J..

[25] Abd-Alla H.I., Ibrahim M.T., Taie H.A.A., Hasan M.A., Shalaby N.M. (2020). Antioxidant and the Efficacy of Sophora secundiflora and Methoxyisoflavones in the Immune Function of Pigeons Vaccinated against Paramyxovirus Serotype-1. Pharmacogn. J..

[26] Kshirsagar S.G., Rao R.V. (2021). Antiviral and immunomodulation effects of Artemisia. Medicina.

[27] Atawodi S.E., Atawodi J.C. (2009). Azadirachta indica (neem): A plant of multiple biological and pharmacological activities. Phytochem. Rev..

[28] Wafaa A., Howaida I., Hassan A., El-Safty M. (2007). Chemical composition and’in vitro’antiviral activity of Azadirachta indica A. Juss (neem) leaves and fruits against newcastle disease virus and infectious bursal disease virus. Aust. J. Basic Appl. Sci..

[29] Aziz W.M., Hamed M.A., Abd-Alla H.I., Ahmed S.A. (2022). Pulicaria crispa mitigates nephrotoxicity induced by carbon tetrachloride in rats via regulation oxidative, inflammatory, tubular and glomerular indices. Biomarkers.

[30] Yu M.-S., Lee J., Lee J.M., Kim Y., Chin Y.-W., Jee J.-G., Keum Y.-S., Jeong Y.-J. (2012). Identification of myricetin and scutellarein as novel chemical inhibitors of the SARS coronavirus helicase, nsP13. Bioorganic Med. Chem. Lett..

[31] Awad H.M., Abd-Alla H.I., Mahmoud K.H., El-Toumy S.A. (2014). In vitro anti-nitrosative, antioxidant, and cytotoxicity activities of plant flavonoids: A comparative study. Med. Chem. Res..

[32] Guo G., Ye L., Pan K., Chen Y., Xing D., Yan K., Chen Z., Ding N., Li W., Huang H. (2020). New insights of emerging SARS-CoV-2: Epidemiology, etiology, clinical features, clinical treatment, and prevention. Front. Cell Dev. Biol..

[33] Elebeedy D., Elkhatib W.F., Kandeil A., Ghanem A., Kutkat O., Alnajjar R., Saleh M.A., Abd El Maksoud A.I., Badawy I., Al-Karmalawy A.A. (2021). Anti-SARS-CoV-2 activities of tanshinone IIA, carnosic acid, rosmarinic acid, salvianolic acid, baicalein, and glycyrrhetinic acid between computational and in vitro insights. RSC Adv..

[34] El-Shershaby M.H., Ghiaty A., Bayoumi A.H., Al-Karmalawy A.A., Husseiny E.M., El-Zoghbi M.S., Abulkhair H.S. (2021). From triazolophthalazines to triazoloquinazolines: A bioisosterism-guided approach toward the identification of novel PCAF inhibitors with potential anticancer activity. Bioorganic Med. Chem..

[35] Mahmoud A., Mostafa A., Al-Karmalawy A.A., Zidan A., Abulkhair H.S., Mahmoud S.H., Shehata M., Elhefnawi M.M., Ali M.A. (2021). Telaprevir is a potential drug for repurposing against SARS-CoV-2: Computational and <em>in vitro</em> studies. Heliyon.

[36] Abd-Alla H.I., Hassan A.Z., Soltan M.M., Abdelwahab A.B., Hanna A.G. (2021). Potential protein antiglycation, antiproliferation, and in silico study on the antidiabetic enzymes of bioactive metabolites from Adonis microcarpa DC and their ADMET properties. J. Appl. Pharm. Sci..

[37] Soltan M.A., Elbassiouny N., Gamal H., Elkaeed E.B., Eid R.A., Eldeen M.A., Al-Karmalawy A.A. (2021). In Silico Prediction of a Multitope Vaccine against Moraxella catarrhalis: Reverse Vaccinology and Immunoinformatics. Vaccines.

[38] El-Shershaby M.H., El-Gamal K.M., Bayoumi A.H., El-Adl K., Alswah M., Ahmed H.E.A., Al-Karmalamy A.A., Abulkhair H.S. (2021). The antimicrobial potential and pharmacokinetic profiles of novel quinoline-based scaffolds: Synthesis and in silico mechanistic studies as dual DNA gyrase and DHFR inhibitors. New J. Chem..

[39] Abd-Alla H.I., Soltan M.M., Hassan A.Z., Taie H.A., Abo-Salem H.M., Karam E.A., El-Safty M.M., Hanna A.G. (2021). Cardenolides and pentacyclic triterpenes isolated from Acokanthera oblongifolia leaves: Their biological activities with molecular docking study. Z. Für Nat. C.

[40] Norrehed S., Johansson H., Grennberg H., Gogoll A. (2013). Improved stereochemical analysis of conformationally flexible diamines by binding to a bisporphyrin molecular clip. Chem. –A Eur. J..

[41] Duddeck H., Kaiser M. (1982). 13C NMR spectroscopy of coumarin derivatives. Org. Magn. Reson..

[42] Shalaby N.M., Abd-Alla H.I., Aly H.F., Albalawy M.A., Shaker K.H., Bouajila J. (2014). Preliminary in vitro and in vivo evaluation of antidiabetic activity of Ducrosia anethifolia Boiss. and its linear furanocoumarins. BioMed Res. Int..

[43] Harborne J., Mabry T. (1982). The Flavonoids: Advances in Research.

[44] Agrawal P.K. (2013). Carbon-13 NMR of flavonoids.

[45] Anton R., Jiang Y., Weniger B., Beck J., Rivier L. (1993). Pharmacognosy of Mimosa tenuiflora (willd.) poiret. J. Ethnopharmacol..

[46] Faizi S., Ali M., Saleem R., Bibi S. (2001). Complete 1H and 13C NMR assignments of stigma-5-en-3-O-β-glucoside and its acetyl derivative. Magn. Reson. Chem..

[47] Quradha M.M., Khan R., Adhikari A., Rauf A., Rashid U., Bawazeer S., Al-Awthan Y.S., Bahattab O., Mubarak M.S. (2021). Isolation, biological evaluation, and molecular docking studies of compounds from Sophora mollis (Royle) Graham Ex Baker. ACS Omega.

[48] Mosmann T. (1983). Rapid colorimetric assay for cellular growth and survival: Application to proliferation and cytotoxicity assays. J. Immunol. Methods.

[49] Hayden F.G., Cote K., Douglas Jr R.G. (1980). Plaque inhibition assay for drug susceptibility testing of influenza viruses. Antimicrob. Agents Chemother..

[50] Wu P., Ma G., Li N., Deng Q., Yin Y., Huang R. (2015). Investigation of in vitro and in vivo antioxidant activities of flavonoids rich extract from the berries of Rhodomyrtus tomentosa (Ait.) Hassk. Food Chem..

[51] Abd-Alla H.I., Souguir D., Radwan M.O. (2021). Genus Sophora: A comprehensive review on secondary chemical metabolites and their biological aspects from past achievements to future perspectives. Arch. Pharmacal Res..

[52] Boozari M., Soltani S., Iranshahi M. (2019). Biologically active prenylated flavonoids from the genus Sophora and their structure–activity relationship—A review. Phytother. Res..

[53] Zhang S., Liang Q., He X., Zhao C., Ren W., Yang Z., Wang Z., Ding Q., Deng H., Wang T. (2022). Loss of Spike N370 glycosylation as an important evolutionary event for the enhanced infectivity of SARS-CoV-2. Cell Res..

[54] Zhang L., Lin D., Sun X., Curth U., Drosten C., Sauerhering L., Becker S., Rox K., Hilgenfeld R. (2020). Crystal structure of SARS-CoV-2 main protease provides a basis for design of improved α-ketoamide inhibitors. Science.

[55] Tobita K. (1975). Permanent canine kidney (MDCK) cells for isolation and plaque assay of influenza B viruses. Med. Microbiol. Immunol..

[56] Alnajjar R., Mostafa A., Kandeil A., Al-Karmalawy A.A. (2020). Molecular docking, molecular dynamics, and in vitro studies reveal the potential of angiotensin II receptor blockers to inhibit the COVID-19 main protease. Heliyon.

[57] Elebeedy D., Badawy I., Elmaaty A.A., Saleh M.M., Kandeil A., Ghanem A., Kutkat O., Alnajjar R., Abd El Maksoud A.I., Al-Karmalawy A.A. (2022). In vitro and computational insights revealing the potential inhibitory effect of Tanshinone IIA against influenza A virus. Comput. Biol. Med..

[58] Kuo Y.C., Lin L.C., Tsai W.J., Chou C.J., Kung S.H., Ho Y.H. (2002). Samarangenin B from Limonium sinense suppresses herpes simplex virus type 1 replication in Vero cells by regulation of viral macromolecular synthesis. Antimicrob. Agents Chemother..

[59] Harcourt J., Tamin A., Lu X., Kamili S., Sakthivel S.K., Murray J., Queen K., Tao Y., Paden C.R., Zhang J. (2020). Severe acute respiratory syndrome coronavirus 2 from patient with coronavirus disease, United States. Emerg. Infect. Dis..

[60] Schuhmacher A., Reichling J., Schnitzler P. (2003). Virucidal effect of peppermint oil on the enveloped viruses herpes simplex virus type 1 and type 2 in vitro. Phytomed. Int. J. Phytother. Phytopharm..

[61] Kandeil A., Mostafa A., Kutkat O., Moatasim Y., Al-Karmalawy A.A., Rashad A.A., Kayed A.E., Kayed A.E., El-Shesheny R., Kayali G. (2021). Bioactive Polyphenolic Compounds Showing Strong Antiviral Activities against Severe Acute Respiratory Syndrome Coronavirus 2. Pathogens.

[62] Chemical Computing Group Inc. (2016). Molecular Operating Environment (MOE).

[63] Mahmoud D.B., Bakr M.M., Al-Karmalawy A.A., Moatasim Y., El Taweel A., Mostafa A. (2022). Scrutinizing the feasibility of nonionic surfactants to form isotropic bicelles of curcumin: A potential antiviral candidate against COVID-19. AAPS PharmSciTech.

[64] Elmaaty A.A., Darwish K.M., Chrouda A., Boseila A.A., Tantawy M.A., Elhady S.S., Shaik A.B., Mustafa M., Al-karmalawy A.A. (2022). In Silico and In Vitro Studies for Benzimidazole Anthelmintics Repurposing as VEGFR-2 Antagonists: Novel Mebendazole-Loaded Mixed Micelles with Enhanced Dissolution and Anticancer Activity. ACS Omega.

[65] Hazem R.M., Antar S.A., Nafea Y.K., Al-Karmalawy A.A., Saleh M.A., El-Azab M.F. (2022). Pirfenidone and vitamin D mitigate renal fibrosis induced by doxorubicin in mice with Ehrlich solid tumor. Life Sci..

[66] Hamed M.I.A., Darwish K.M., Soltane R., Chrouda A., Mostafa A., Abo Shama N.M., Elhady S.S., Abulkhair H.S., Khodir A.E., Elmaaty A.A. (2021). β-Blockers bearing hydroxyethylamine and hydroxyethylene as potential SARS-CoV-2 Mpro inhibitors: Rational based design, in silico, in vitro, and SAR studies for lead optimization. RSC Adv..

[67] Elia S.G., Al-Karmalawy A.A., Nasr M.Y., Elshal M.F. (2022). Loperamide potentiates doxorubicin sensitivity in triple-negative breast cancer cells by targeting MDR1 and JNK and suppressing mTOR and Bcl-2: In vitro and molecular docking study. J. Biochem. Mol. Toxicol..

[68] Diab R.T., Abdel-Sami Z.K., Abdel-Aal E.H., Al-Karmalawy A.A., Abo-Dya N.E. (2021). Design and synthesis of a new series of 3,5-disubstituted-1,2,4-oxadiazoles as potential colchicine binding site inhibitors: Antiproliferative activity, molecular docking, and SAR studies. New J. Chem..

[69] Raslan M.A.F., Taher R., Al-Karmalawy A.A., El-Ebeedy D., Metwaly A.G., Elkateeb N.M., Ghanem A., Elghaish R.A., Abd El Maksoud, A.I. (2021). *Cordyline fruticosa* (L.) A. Chev. leaves: Isolation, HPLC/MS profiling and evaluation of nephroprotective and hepatoprotective activities supported by molecular docking. New J. Chem..

[70] Taher R.F., Al-Karmalawy A.A., Abd El Maksoud A.I., Khalil H., Hassan A., El-Khrisy E.-D.A., El-Kashak W. (2021). Two new flavonoids and anticancer activity of Hymenosporum flavum: In vitro and molecular docking studies. J Herbmed Pharm..

[71] Mahmoud D.B., Ismail W.M., Moatasim Y., Kutkat O., ElMeshad A.N., Ezzat S.M., El Deeb K.S., El-Fishawy A.M., Gomaa M.R., Kandeil A. (2021). Delineating a potent antiviral activity of Cuphea ignea extract loaded nano-formulation against SARS-CoV-2: In silico and in vitro studies. J. Drug Deliv. Sci. Technol..

[72] Mansour K.A., Elbermawi A., Al-Karmalawy A.A., Lahloub M.-F., El-Neketi M. (2022). Cytotoxic effects of extracts obtained from plants of the Oleaceae family: Bio-guided isolation and molecular docking of new secoiridoids from Jasminum humile. Pharm. Biol..

[73] Salem M.A., El-Shiekh R.A., Aborehab N.M., Al-Karmalawy A.A., Ezzat S.M., Alseekh S., Fernie A.R. (2022). Metabolomics driven analysis of Nigella sativa seeds identifies the impact of roasting on the chemical composition and immunomodulatory activity. Food Chem..

[74] El-Masry R.M., Al-Karmalawy A.A., Alnajjar R., Mahmoud S.H., Mostafa A., Kadry H.H., Abou-Seri S.M., Taher A.T. (2022). Newly synthesized series of oxoindole–oxadiazole conjugates as potential anti-SARS-CoV-2 agents: In silico and in vitro studies. New J. Chem..

[75] Aljuhani A., Ahmed H.E., Ihmaid S.K., Omar A.M., Althagfan S.S., Alahmadi Y.M., Ahmad I., Patel H., Ahmed S., Almikhlafi M.A. (2022). In vitro and computational investigations of novel synthetic carboxamide-linked pyridopyrrolopyrimidines with potent activity as SARS-CoV-2-M Pro inhibitors. RSC Adv..

