# Supplementary material for: Investigating the Potential Anti-SARS-CoV-2 and Anti-MERS-CoV Activities of Yellow Necklacepod among Three Selected Medicinal Plants: Extraction, Isolation, Identification, In Vitro, Modes of Action, and Molecular Docking Studies
Source: Metabolites. 2022 Nov 13;12(11):1109. doi: 10.3390/metabo12111109 (PMC9696309; doi:10.3390/metabo12111109)
Supplement: Supplementary file 1 [file metabolites-12-01109-s001.zip › metabolites-2024093-supplementary.pdf]

Investigating the Potential Anti-SARS-CoV-2 and Anti-MERS-CoV Activities of Yellow Necklacepod among Three Selected Medicinal Plants: Extraction, Isolation, Identification, *In Vitro*, Modes of Action, and Molecular Docking Studies

Howaida I. Abd-Alla[1,*], Omnia Kutkat[2], Heba-tollah M. Sweelam[1], Wagdy M. Eldehna[3,4], Marwa A. Mostafa[5], Magda T. Ibrahim[6], Yassmin Moatasim[2], Mohamed GabAllah[2], and Ahmed A. Al-Karmalawy[7,*]

[1] Chemistry of Natural Compounds Department, Pharmaceutical and Drug Industries Research Institute, National Research Centre, Dokki-Giza 12622, Egypt.

[2] The Center of Scientific Excellence for Influenza Viruses, Water Pollution Research Department, Environmental Research Institute, National Research Centre, Dokki-Giza 12622, Egypt.

[3] Department of Pharmaceutical Chemistry, Faculty of Pharmacy, Kafrelsheikh University, Kafrelsheikh 33516, Egypt.

[4] School of Biotechnology, Badr University in Cairo, Badr City 11829, Egypt.

[5] Bab El Shaariya University Hospital, Cairo, Egypt.

[6] Department of Pharmacognosy, Faculty of Pharmacy (Girls), Al-Azhar University, Egypt.

[7] Pharmaceutical Chemistry Department, Faculty of Pharmacy, Ahram Canadian University, 6th of October City, Giza 12566, Egypt.

[*] **Corresponding authors:**

Ahmed A. Al-Karmalawy; Email: akarmalawy@acu.edu.eg

Howaida I. Abd-Alla; Email: hi.abdalla@nrc.sci.eg

Supplementary Material

SI 1. Identification of isolated compounds from *S. tomentosa*

Compound 3: R_f: 0.29 (S_1), 0.61 (S_2), m.p. 220 °C, UV λ_{max} (nm): (MeOH): 249, 26 (sh) and 302, (+ NaOMe): 254 and 337 (sh), (+ $AlCl_3$): 250, 261 (sh) and 303, (+$AlCl_3$/ HCl): 250, 262 (sh) and 303, (+ NaOAc): 252, 311 and 336 (+NaOAc / H_3BO_3): 261 and 304(sh).^{1}H-NMR (400 MHz, DMSO-d_6), δ_{ppm} 8.19 (1H, d, *J*= 8.8, H-5), 7.98 (1H, s, H-2),7.57 (2H, d, *J*= 8.8, H-2'/6'), 7.16 (1H, dd, *J*= 2.4 and 8.8, H-6), 7.03 (1H, d, *J*= 2.4, H-8), 6.96 (2H, d, *J*= 8.8, H-3'/5'), 3.52 (3H, s, O-CH_3). ^{13}C NMR (100 MHz, DMSO-d_6), δ_{ppm} 176.6 (C-4), 171.2 (C-7), 159.6 (C-4'), 157.1 (C-9), 152.7 (C-2), 130.02 (C-2'/6'), 126.1 (C-5), 124.6 (C-1'), 123.6 (C-3), 118.2 (C-10), 114.6 (C-6), 114.0 (C-3'/5'), 103.01 (C-8), 54.1 (O-CH_3).

Compound 5: R_f: 0.87 (S_3) and 0.34 (S_4), m.p. 239-241°C; UV λ_{max} (nm): (MeOH): 262 and 327 (sh), (+ NaOMe): 273 and 351, (+ $AlCl_3$): 271, 308 (sh) and 373, (+$AlCl_3$ / HCl): 276, 311 (sh) and 372, (+ NaOAc): 260 and 332, (+NaOAc/H_3BO_3): 261 and 334 (sh).^{1}H-NMR (400 MHz, DMSO-d_6), δ_{ppm} 8.15 (1H, s, H-2), 7.33 (2H, d, *J* = 8.60 Hz, H-2'/6'), 6.83 (2H, d, *J* = 8.60 Hz, H-3'/5'), 6.53 (1H, d, *J* = 2.16 Hz, H-6), 6.30 (1H, d, *J* = 2.16 Hz, H-8), 3.83 (3H, s, O-CH_3). ^{13}C-NMR (100 MHz, DMSO-d_6), δ_{ppm} 180.3 (C-4), 165.2 (C-7), 161.3 (C-5), 157.2 (C-4`), 157.2 (C-9), 153.1 (C-2), 129.8 (C-2'/ 6'), 122.8 (C-3), 121.2 (C-1'), 114.6 (C-3'/5'), 105.3 (C-10), 97.3 (C-6), 91.5 (C-8), 55.06 (O-CH_3)**.**

Compound 9: R_f: 0.49 (S_5), 0.60 (S_4); m.p. 280-282 °C; ^{1}H-NMR ($CDCl_3$, 400 MHz): δppm 5.30 (1 H, brs, H-6), 4.22 (1H, d, *J* = 7.8 Hz, H-1'), 3.58 (1H, m, H-3), 3.42–2.92 (1H, d, H-2'), 2.28 (2H, dd, *J* = 2.5 Hz, 2.5 Hz, H-4), 2.10 (2H, t, *J* = 22.5 Hz, H-7), 1.89 (2H, m, H-15), 1.81 (2 H, dt, *J* = 15.0 Hz, 25.0 Hz, H-16), 1.75 (2H, dt, *J* = 19.0 Hz, *J* = 23.0 Hz, H-2), 1.53 (2H, t, *J* = 10.0 Hz, H-1), 1.47 (1H, m, H-9), 1.36 (1H, m, H-17), 1.38 (2H, t, *J* = 15.0 Hz, H-12), 1.36 (2H, m, H-11), 1.30 (2H, t, *J* = 10.5, H-14), 1.14 (3H, s, H-18), 0.87 (3H, s, H-19), 0.77 (3H, d, *J* = 6.0 Hz, H-21), 0.71 (3H, d, *J* = 10.0 Hz, H-26), 0.68 (3H, t, *J* = 5.0 Hz, H-29), 0.56 (3H, s); ^{13}C-NMR (DMSO-d6, 75MHz): δ ppm 36.6 (C-1), 29.3 (C-2), 78.7 (C-3), 38.9 (C-4), 139.1 (C-5), 121.4 (C-6), 31.2 (C-7/8), 49.9 (C-9), 36.4 (C-10), 21.1 (C-11), 39.9 (C-12/13), 56.5 (C-14), 25.1 (C-15), 26.1 (C-16), 55.8 (C-17), 11.6 (C-18), 19.2 (C-19), 35.9 (C-20), 18.7 (C-21), 33.4 (C-22), 26.8 (C-23), 47.1 (C-24), 29.6 (C-25), 19.7 (C-26), 19.1 (C-27), 22.5 (C-28), 12.1 (C-29), 100.9 (C-1'), 74.5 (C-2'), 76.6 (C-3'/5'), 71.5 (C-4'), 61.0 (C-6').

SI 2. Spectral data of the isolated compounds from *S. tomentosa*

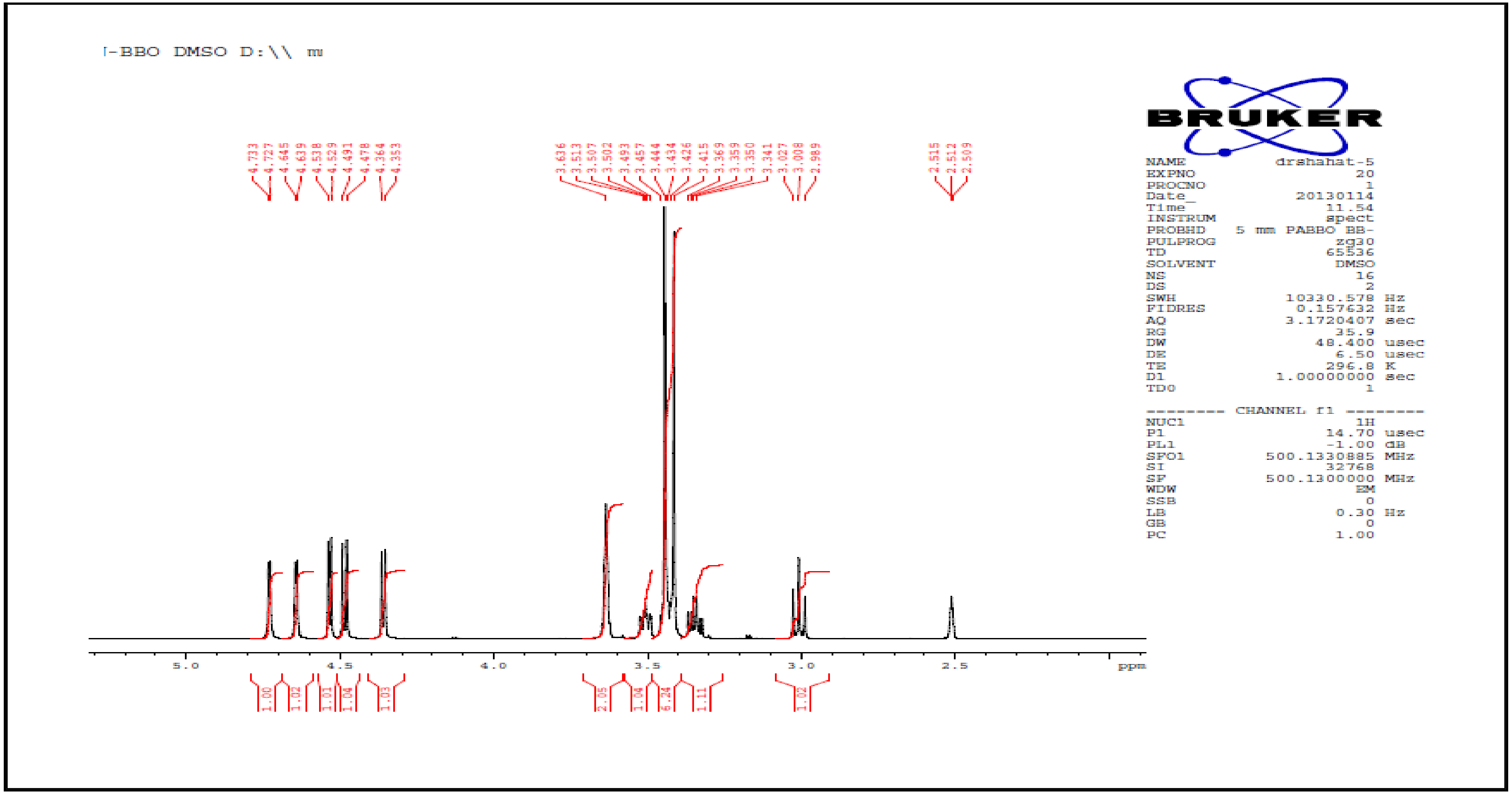

^{1}H-NMR spectrum of compound 1 in DMSO –d$_{6}$

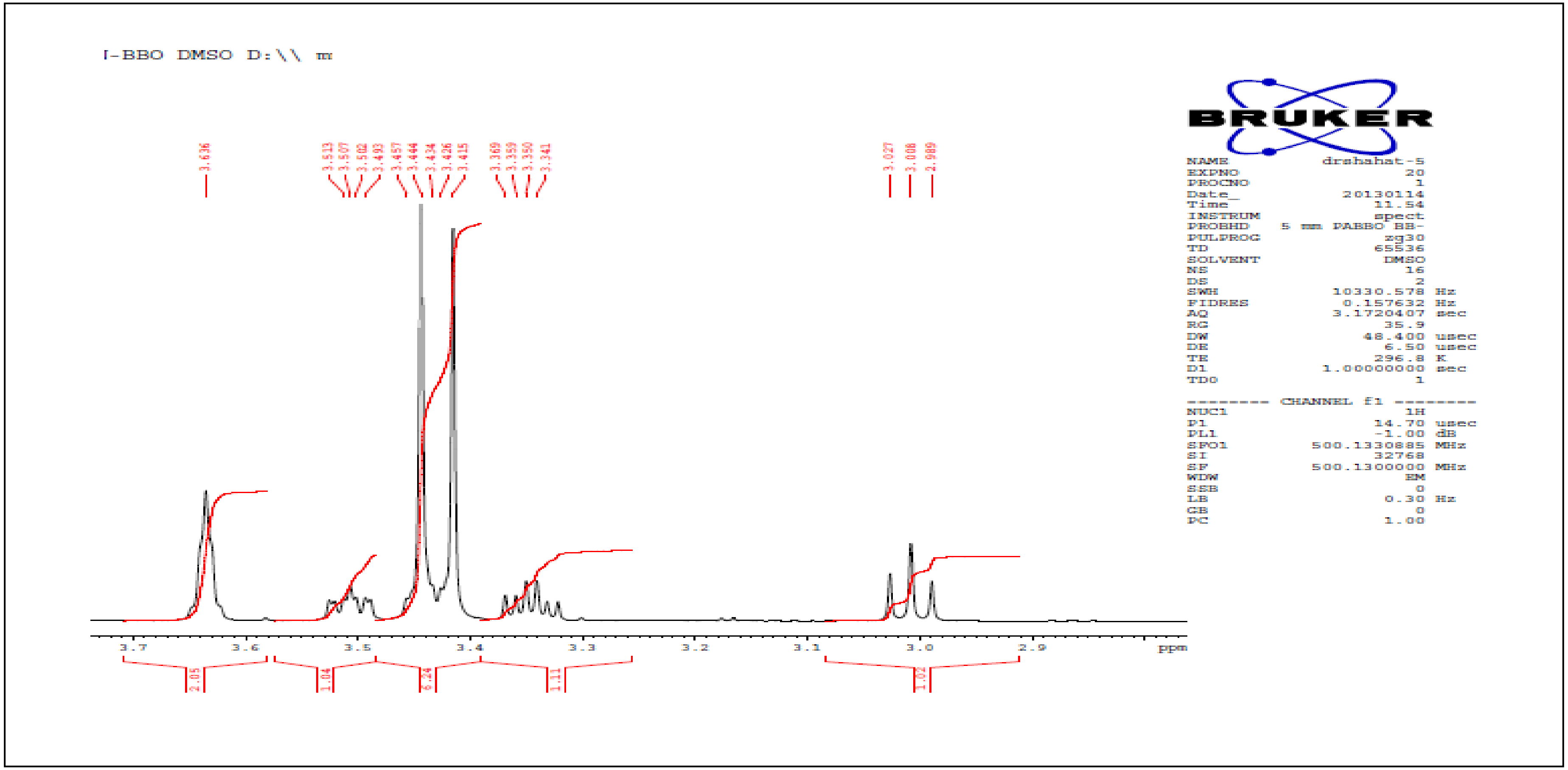

(expansion) ^{1}HNMR spectrum of compound 1 in DMSO –d$_{6}$

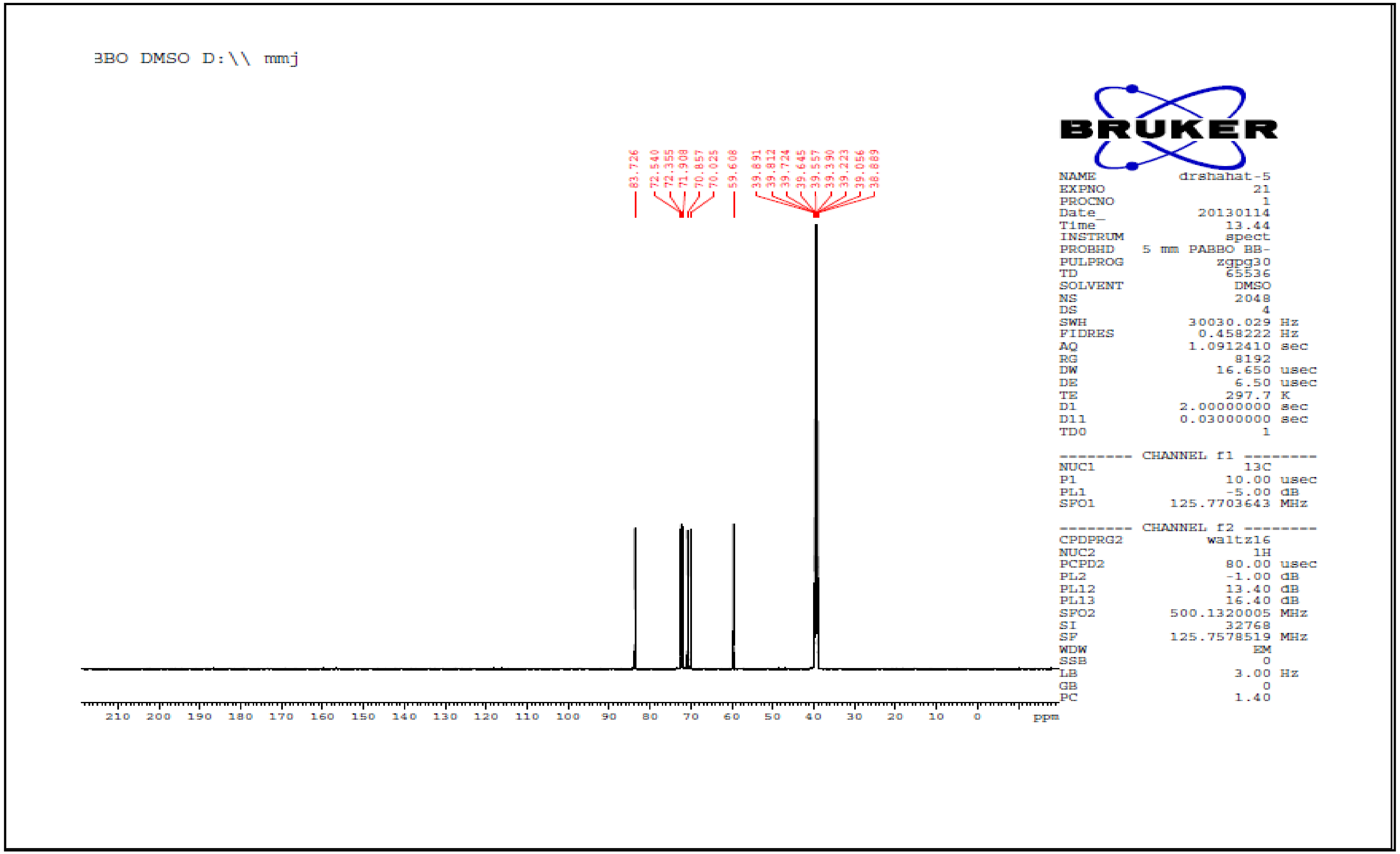

^{13}C-NMR spectrum of compound 1 in DMSO –d_6

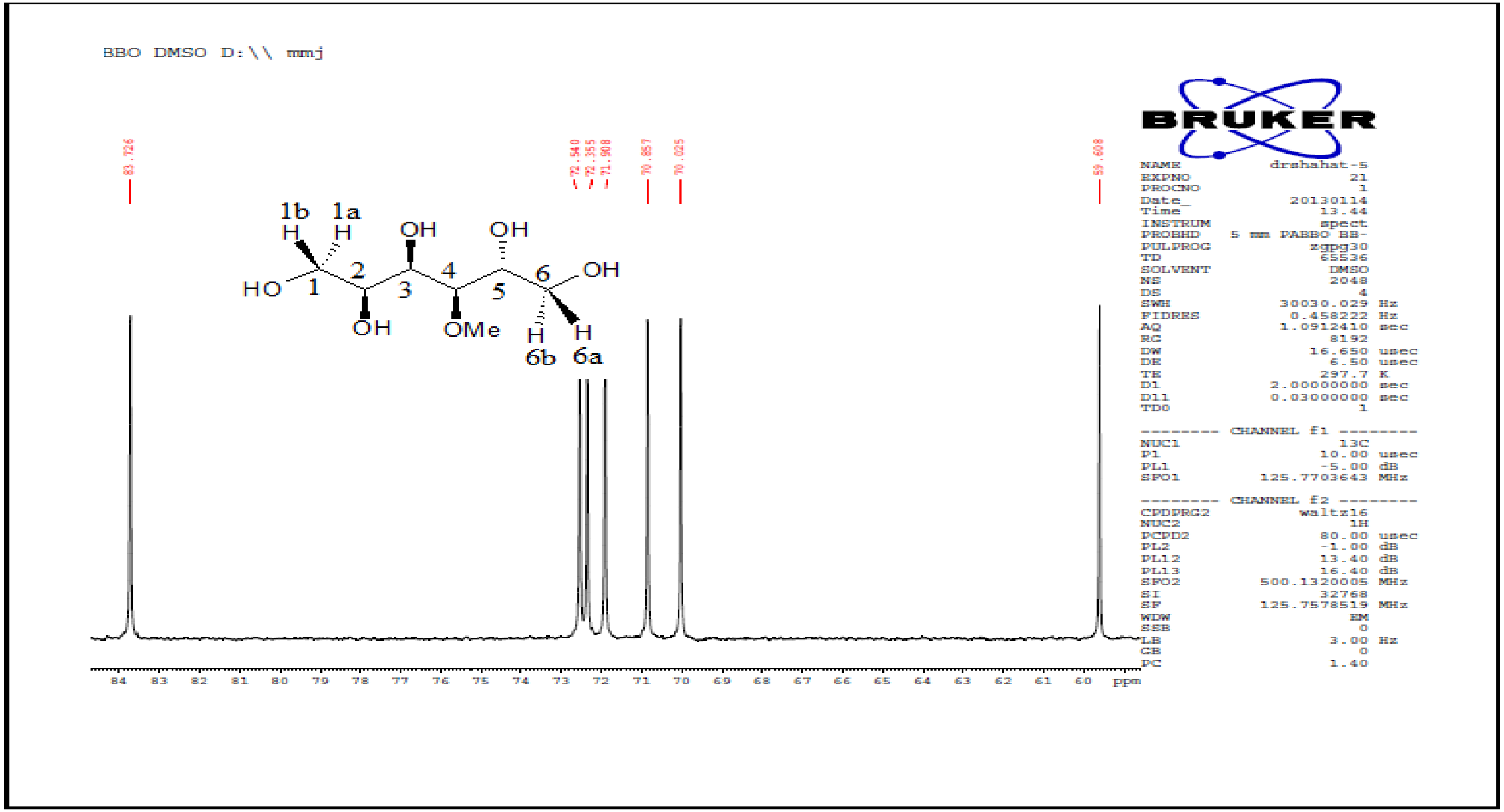

(expansion) ^{13}C NMR spectrum of compound 1 in DMSO –d$_6$

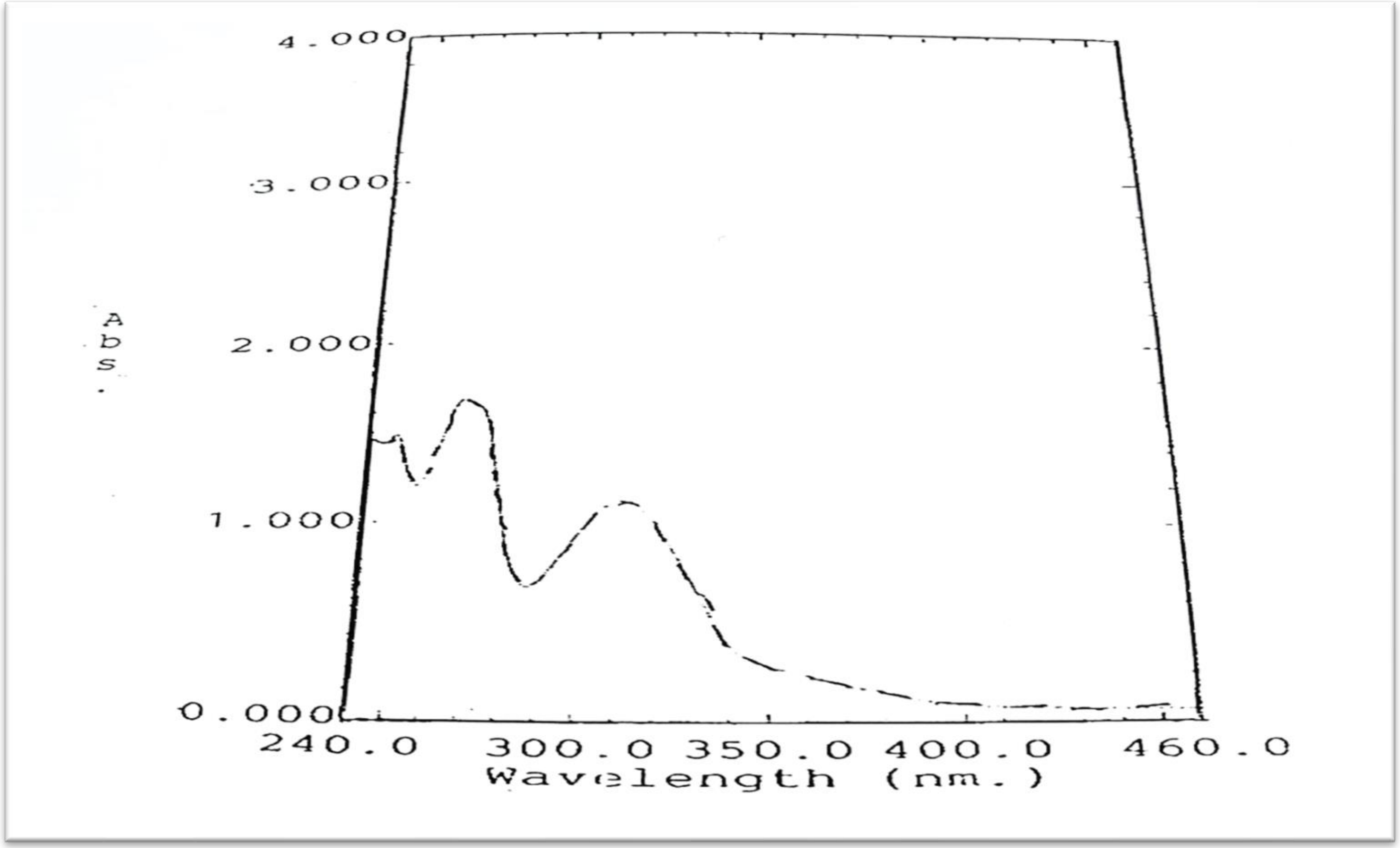

UV spectrum of compound 2 in MeOH

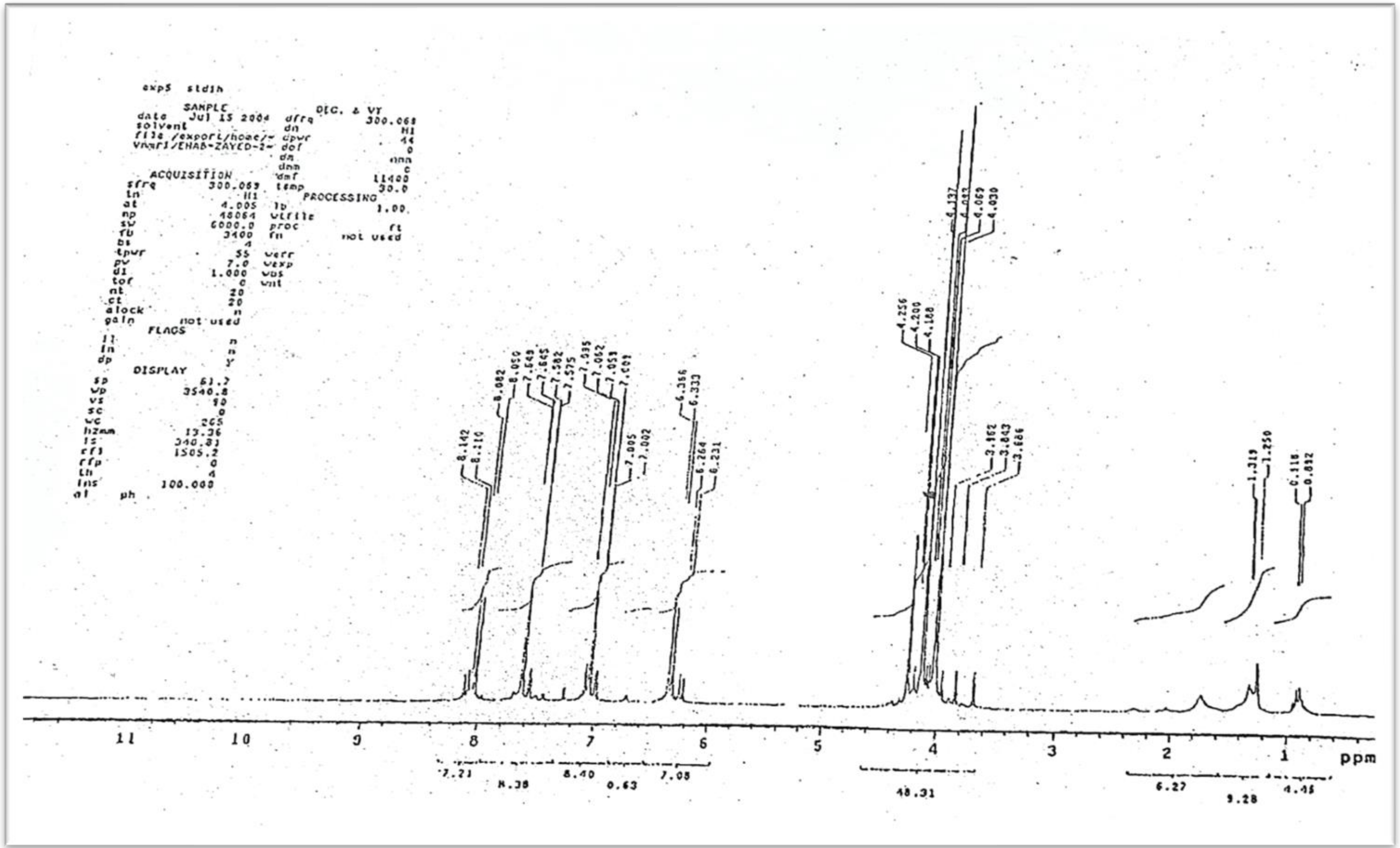

^{1}HNMR spectrum of compound 2 in $CDCl_3$

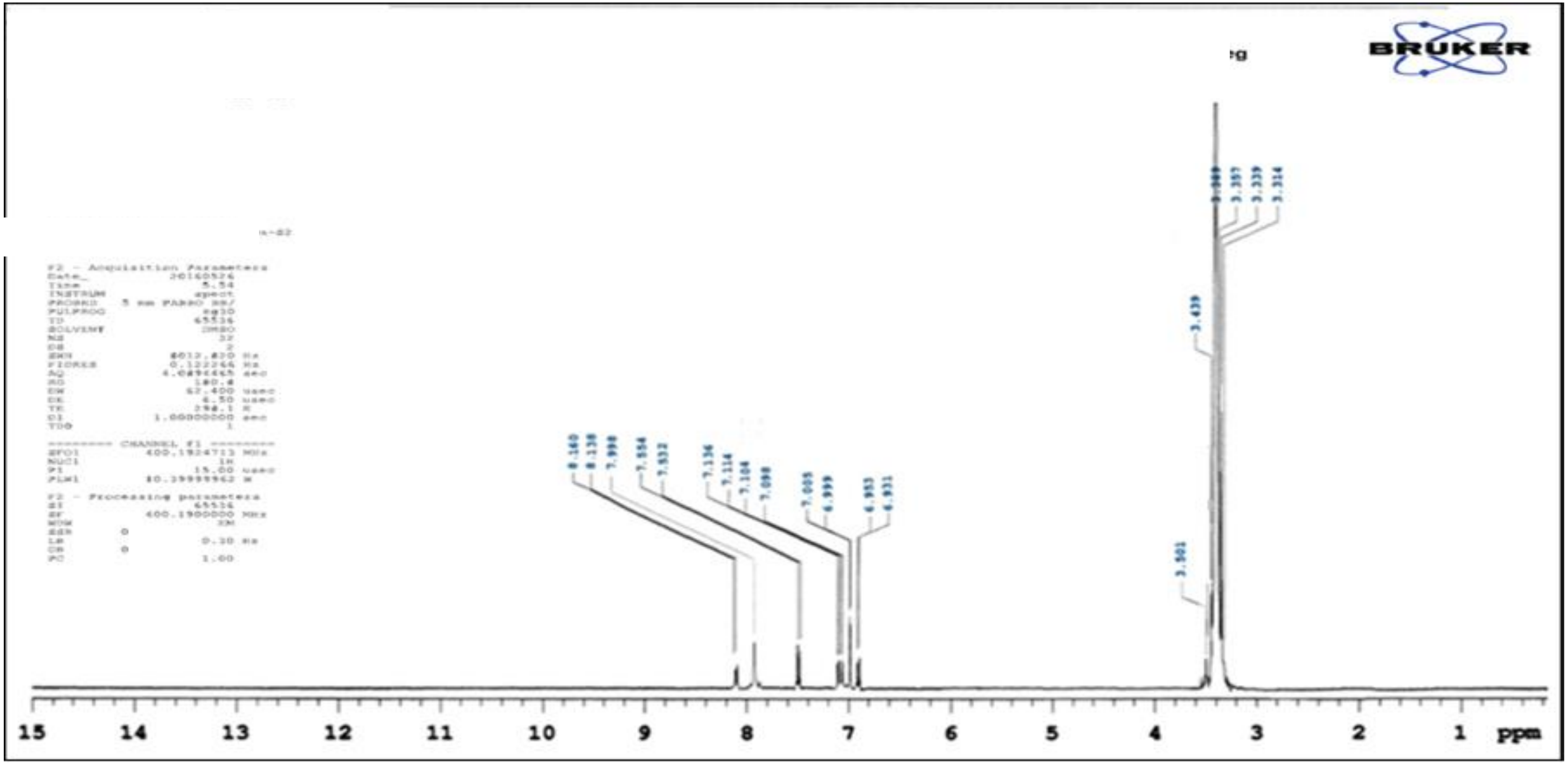

^{1}H-NMR spectrum of compound 3 in DMSO –d_6

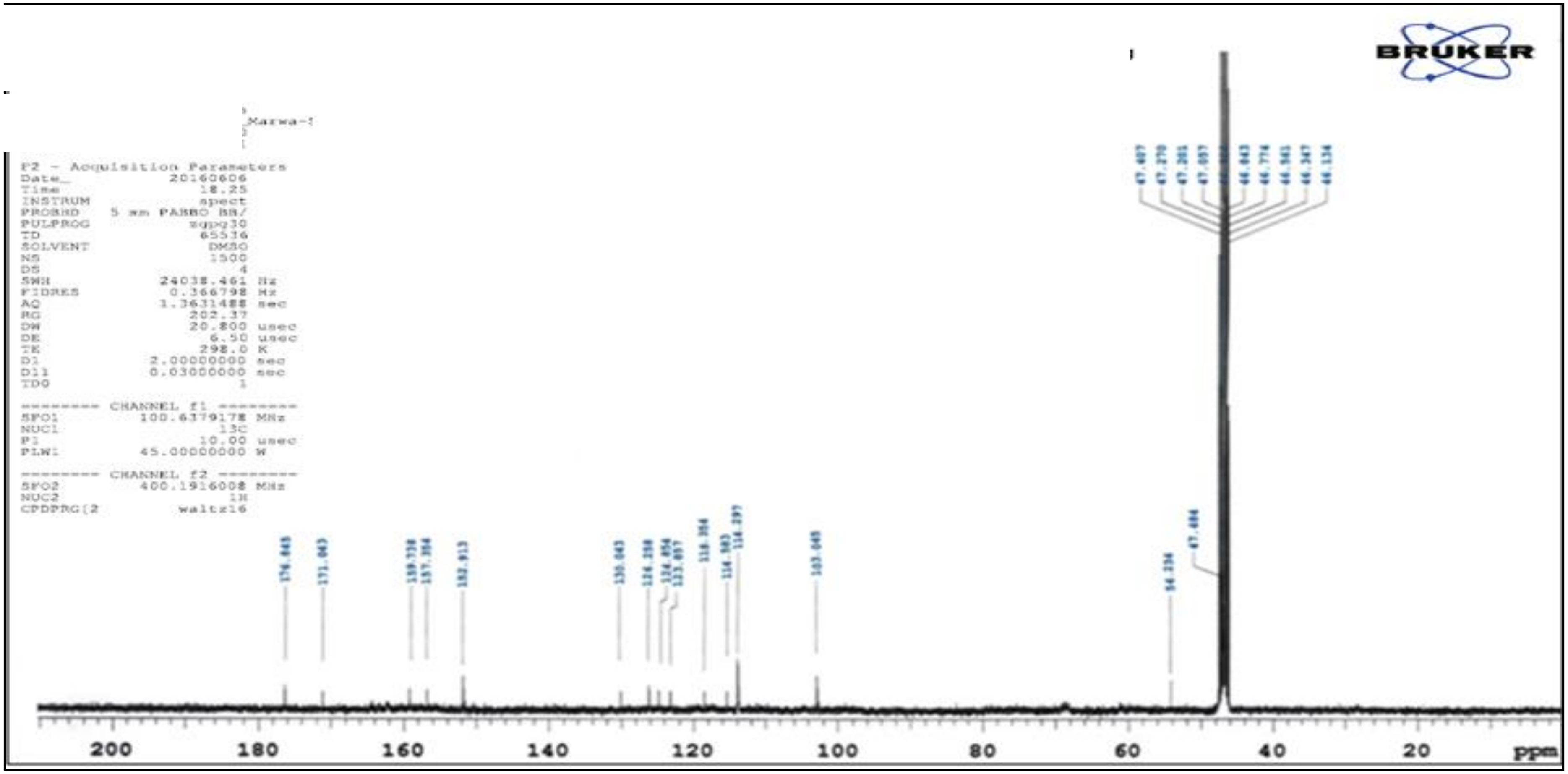

^{13}C-NMR spectrum of compound 3 in DMSO –d$_6$

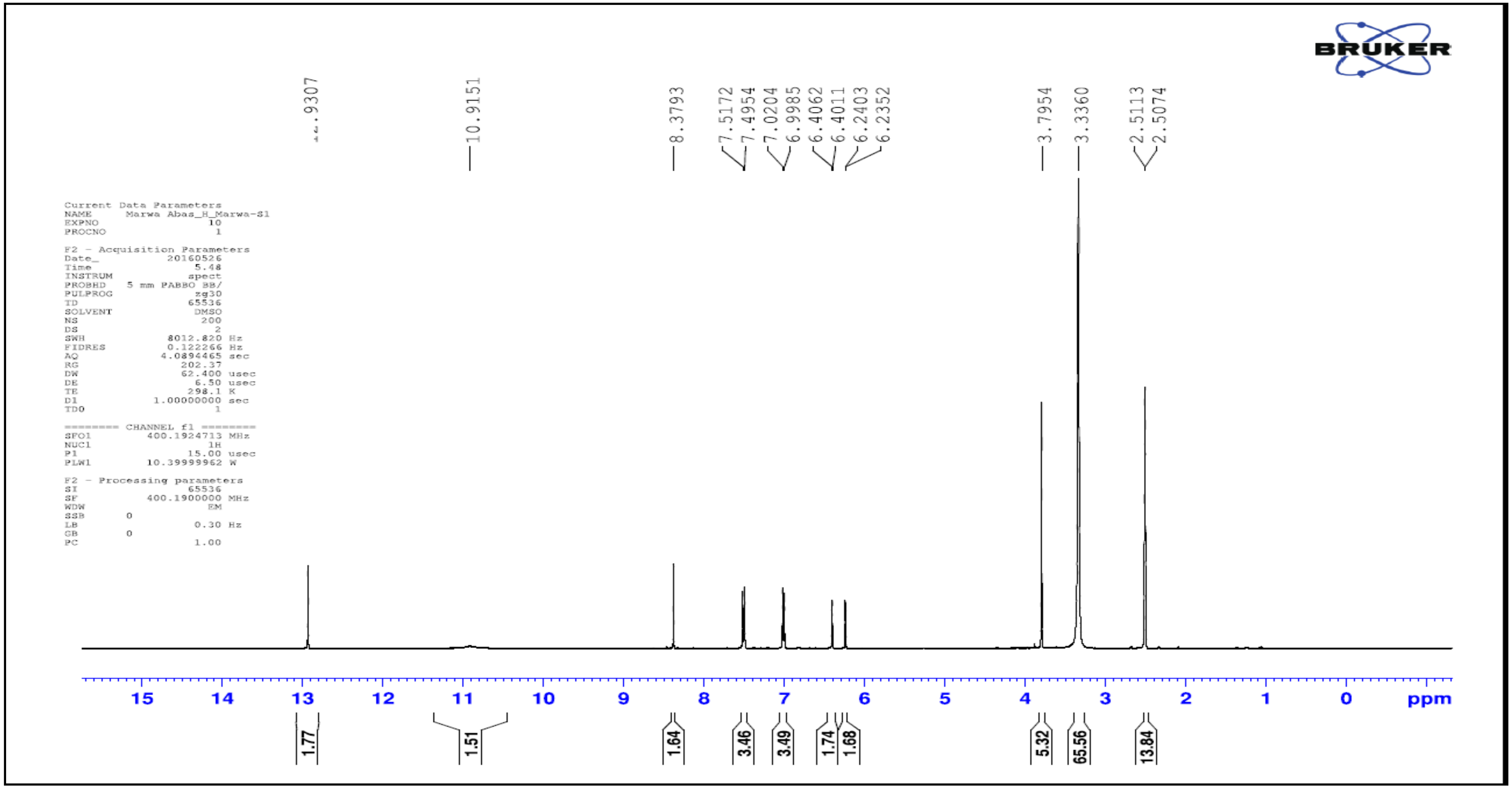

^{1}H-NMR spectrum of compound 4 in DMSO –d$_6$

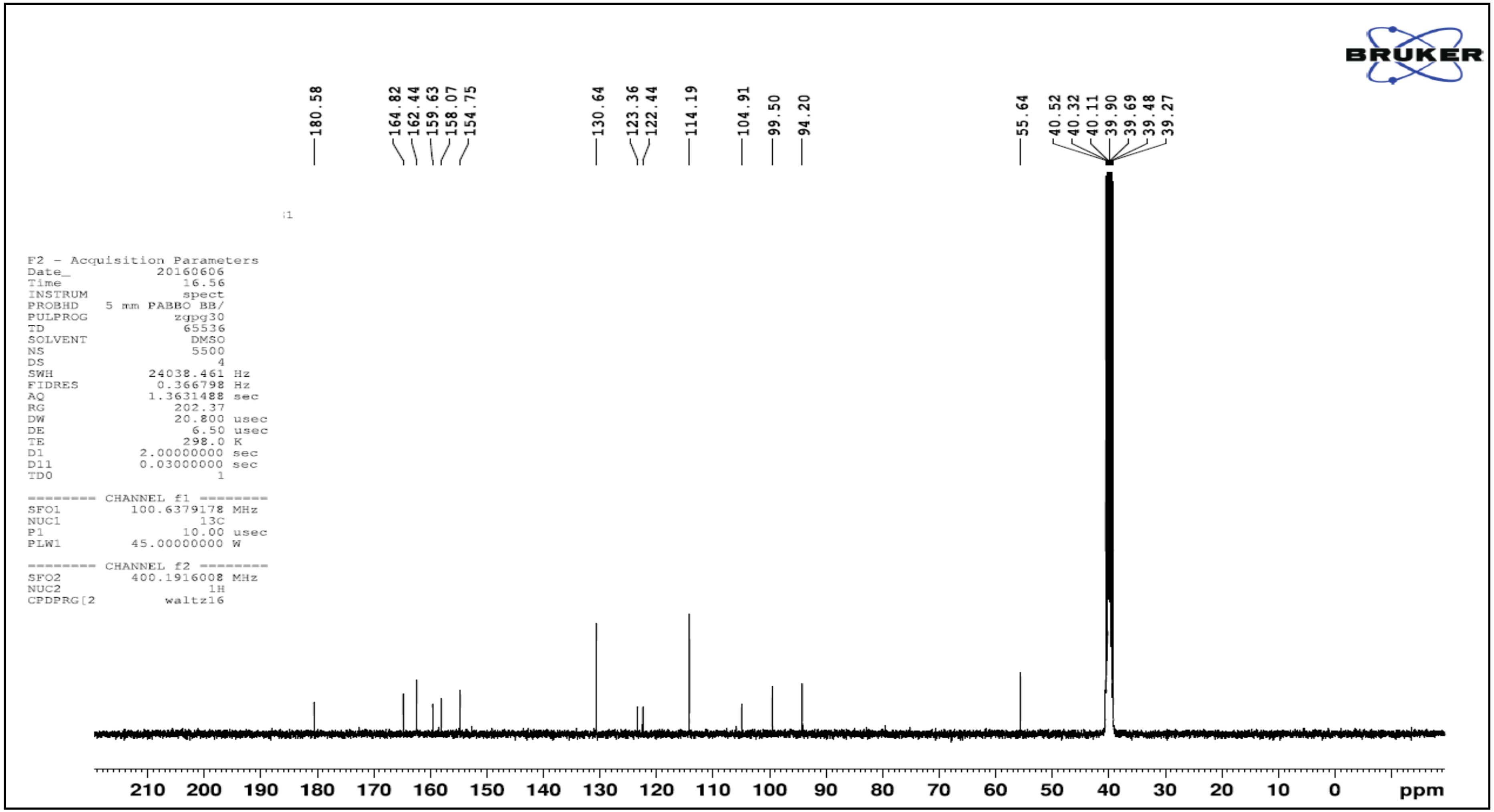

^{13}C-NMR spectrum of compound 4 in DMSO –d_6

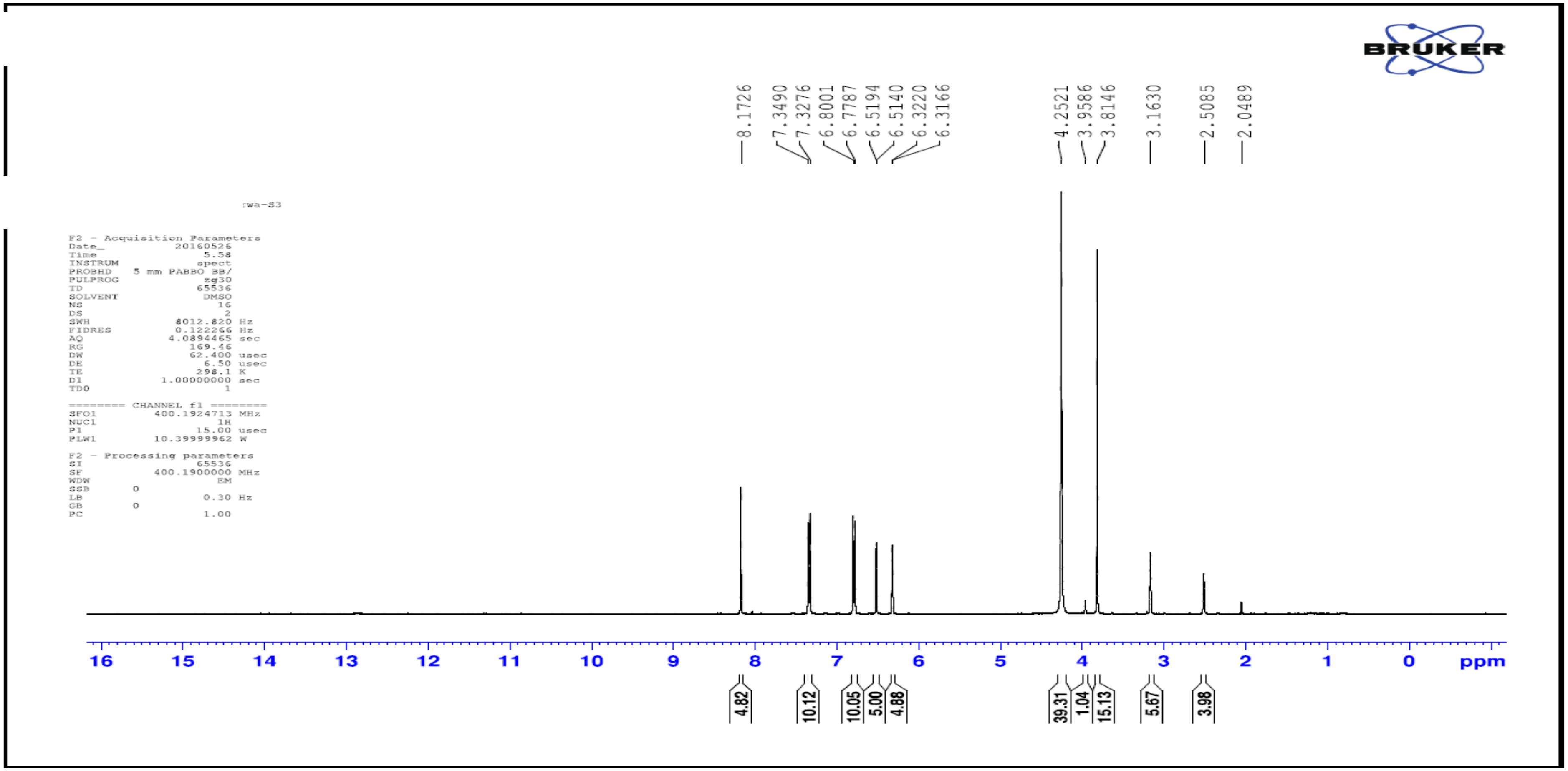

^{1}H-NMR spectrum of compound 5 in DMSO –d_6

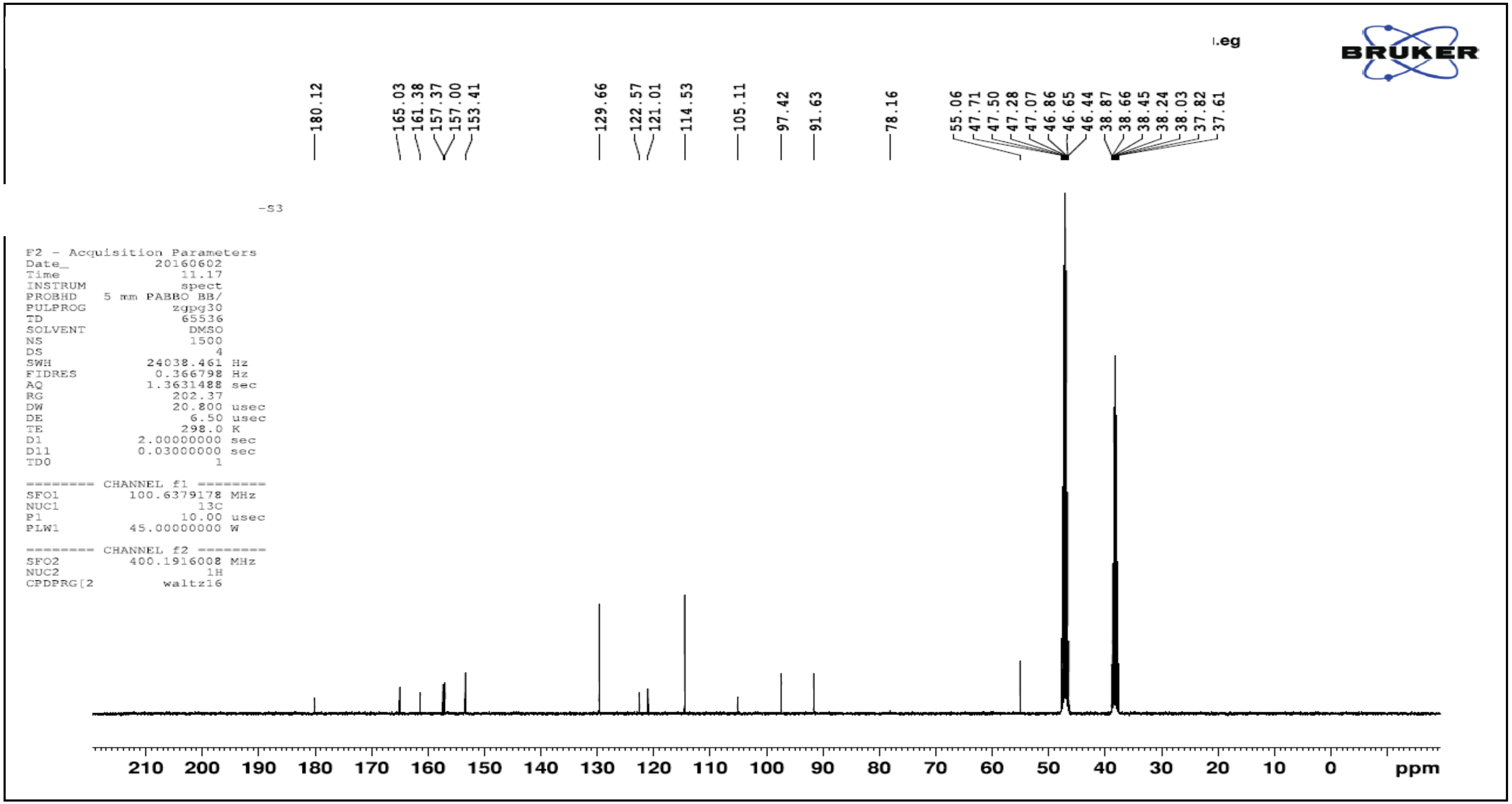

^{13}C-NMR spectrum of compound 5 in DMSO –d_6

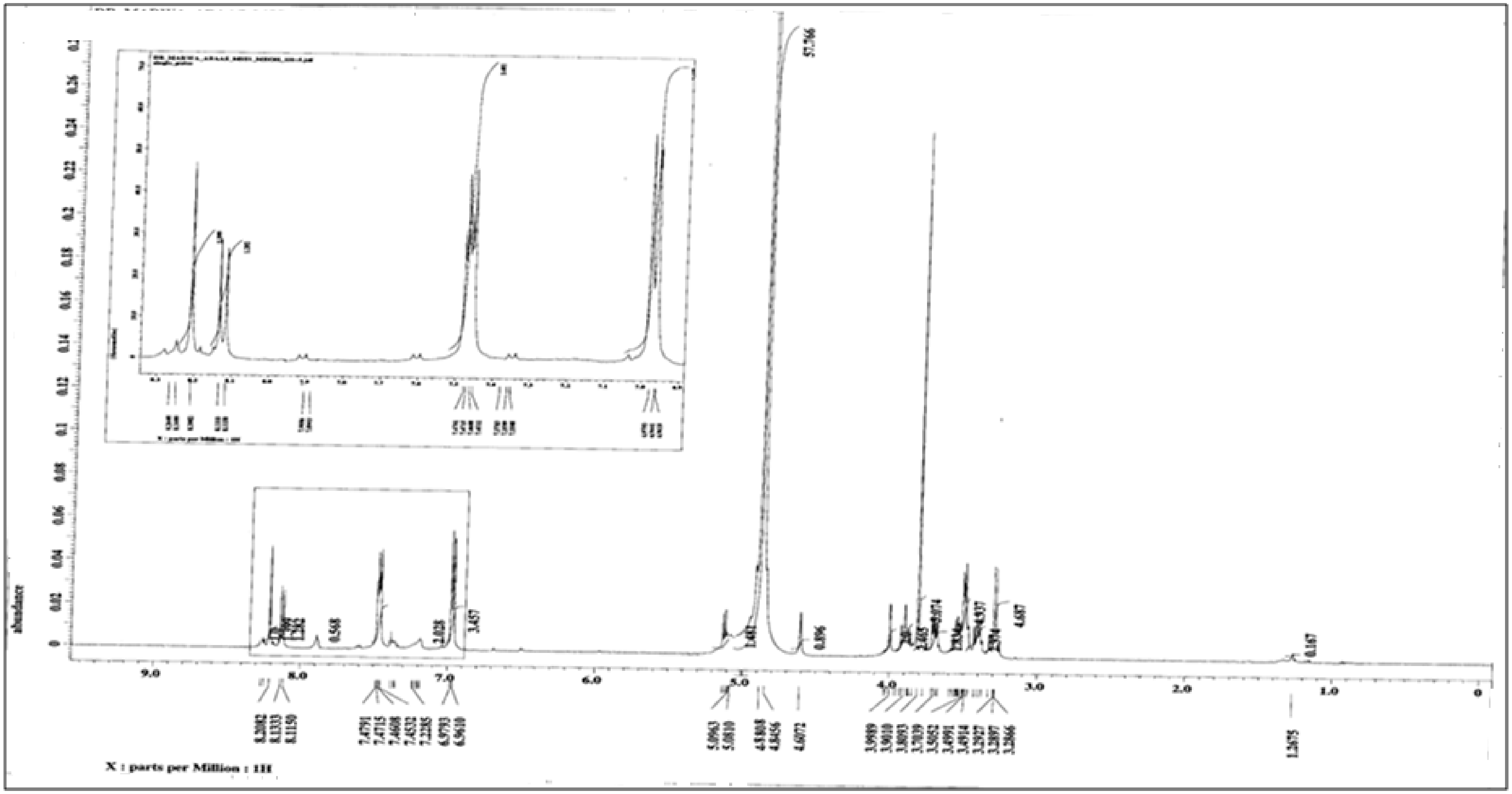

^{1}H-NMR spectrum of compound 6 in DMSO –d_6

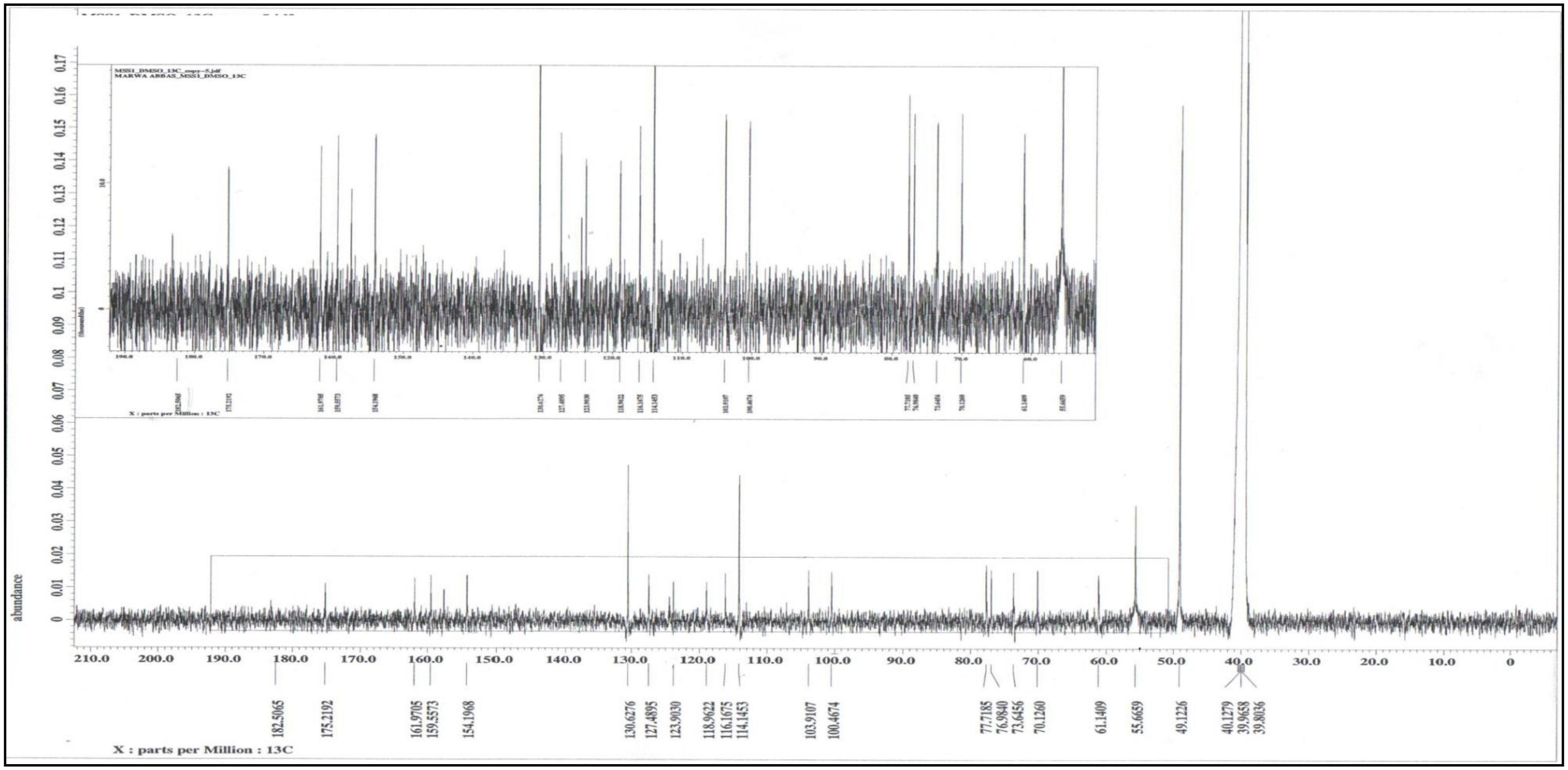

^{13}C-NMR spectrum of compound 6 in DMSO –d_6

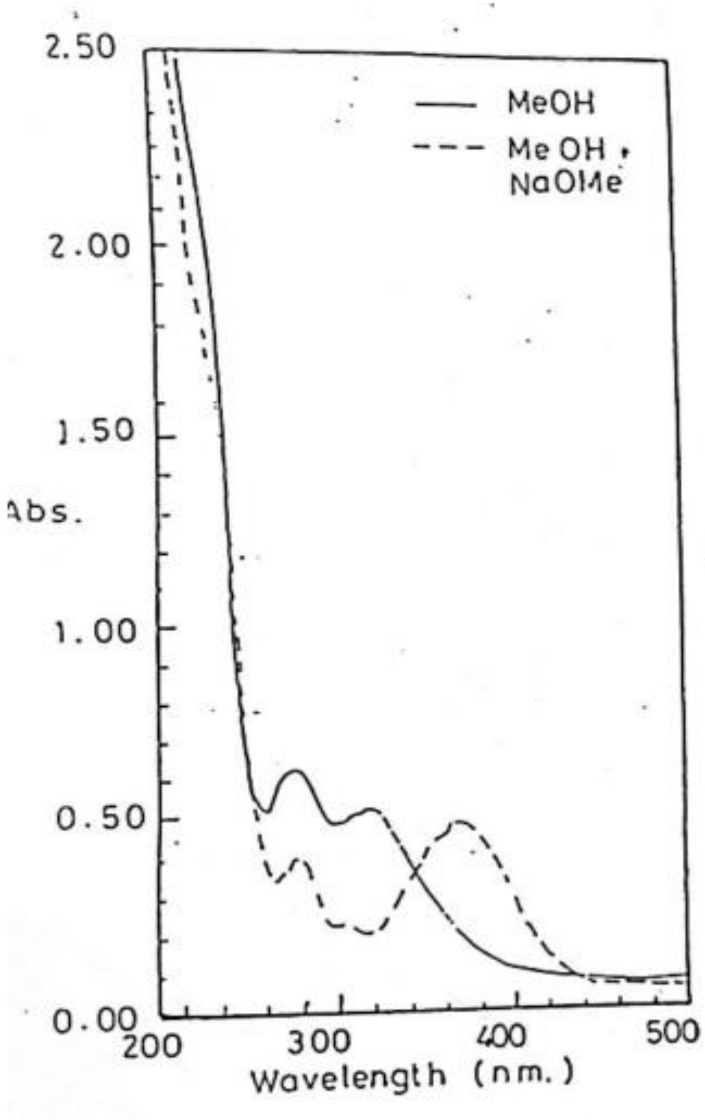
2.50
2.00
1.50
Abs.
1.00
0.50
0.00
200
300
400
500
Wavelength (nm.)
MeOH
MeOH + NaOMe

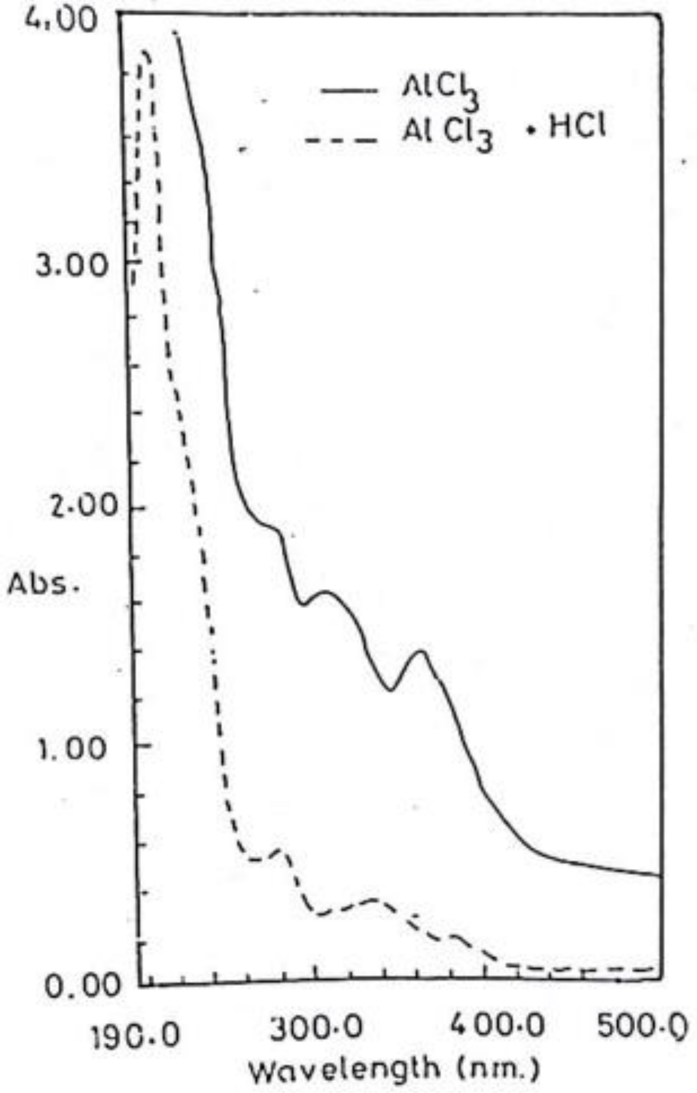
4.00
3.00
2.00
1.00
0.00
Abs.
AlCl3
AlCl3 • HCl
190.0
300.0
400.0
500.0
Wavelength (nm.)

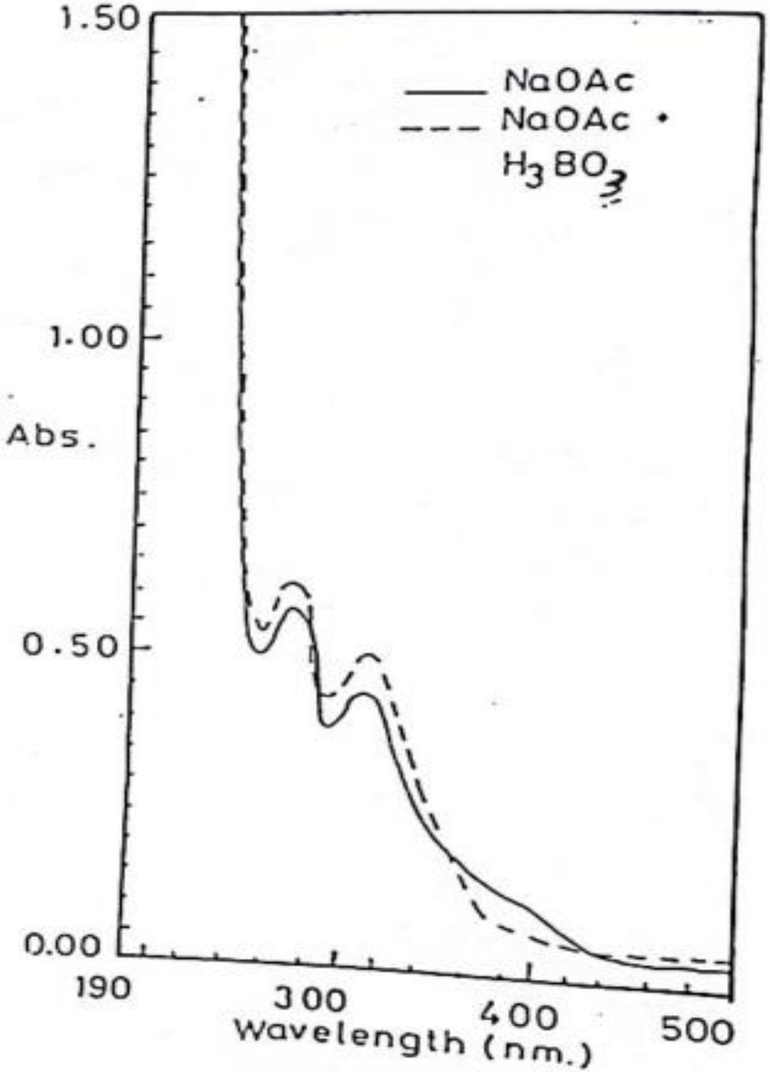
1.50
1.00
0.50
0.00
Abs.
NaOAc
NaOAc +
H_3BO_3
190
300
400
500
Wavelength (nm.)

UV spectrum of compound 7

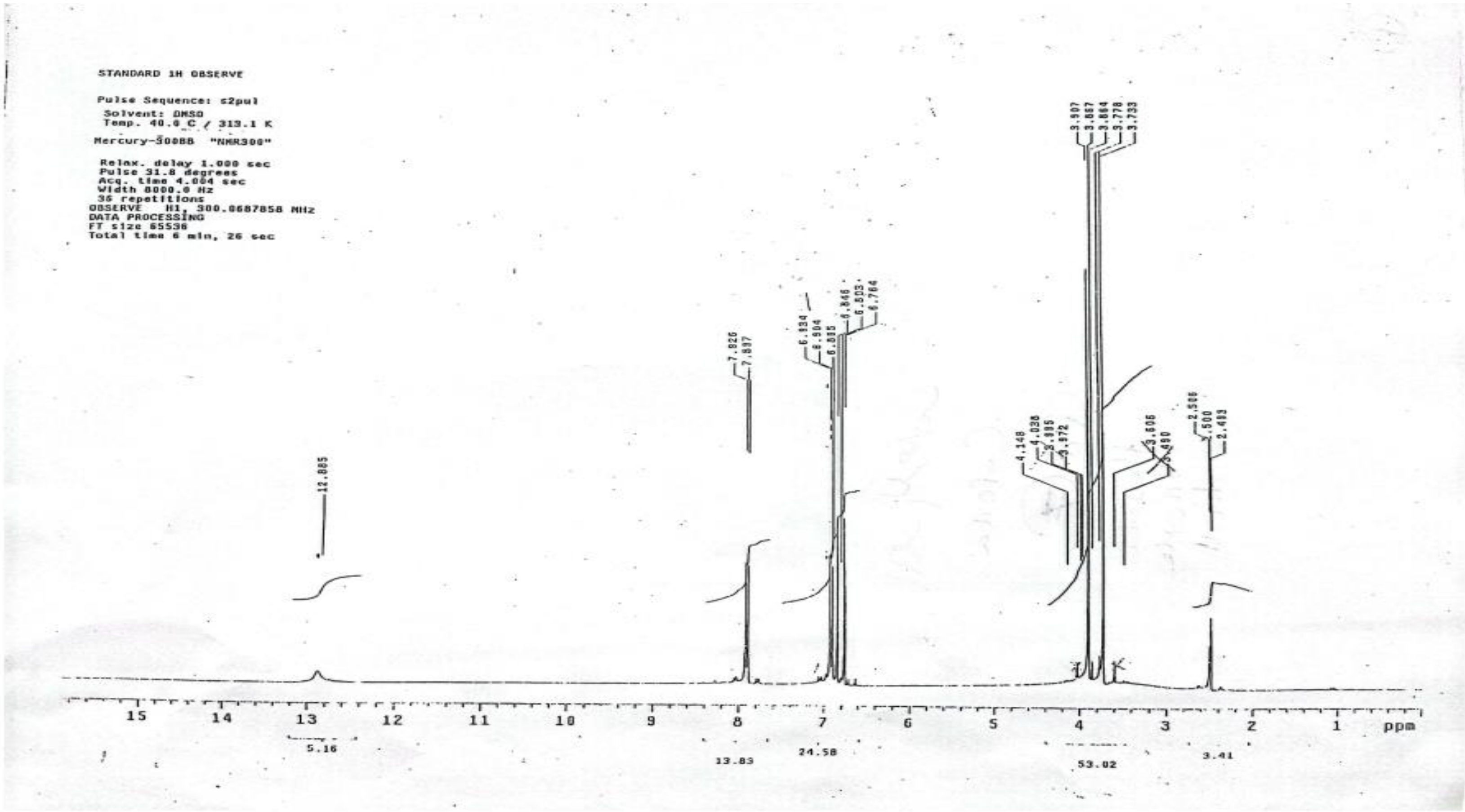

^{1}H NMR spectrum of compound 7 in DMSO –d_6

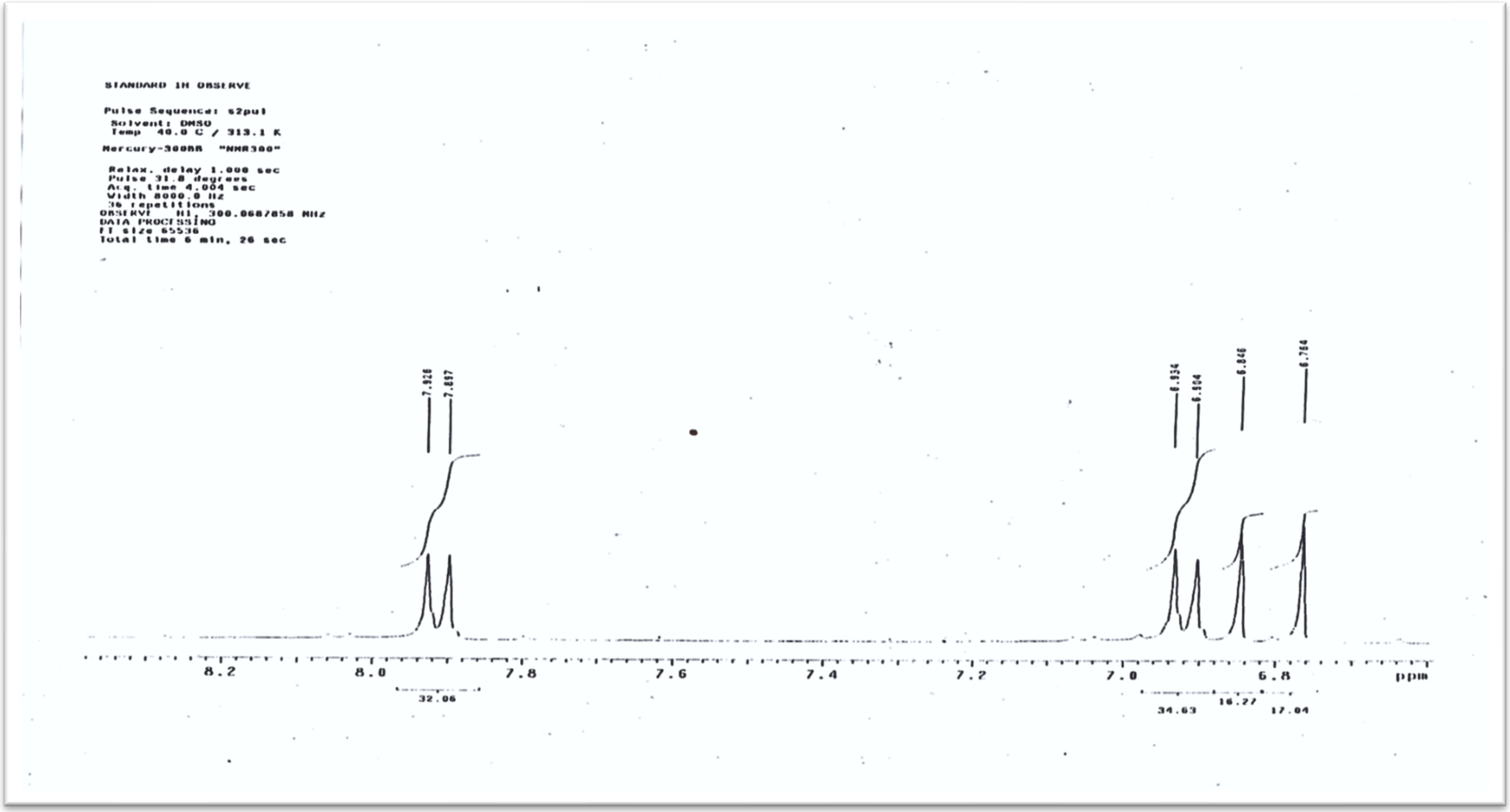

(expansion) ^{1}HNMR spectrum of compound 7 in DMSO –d_6

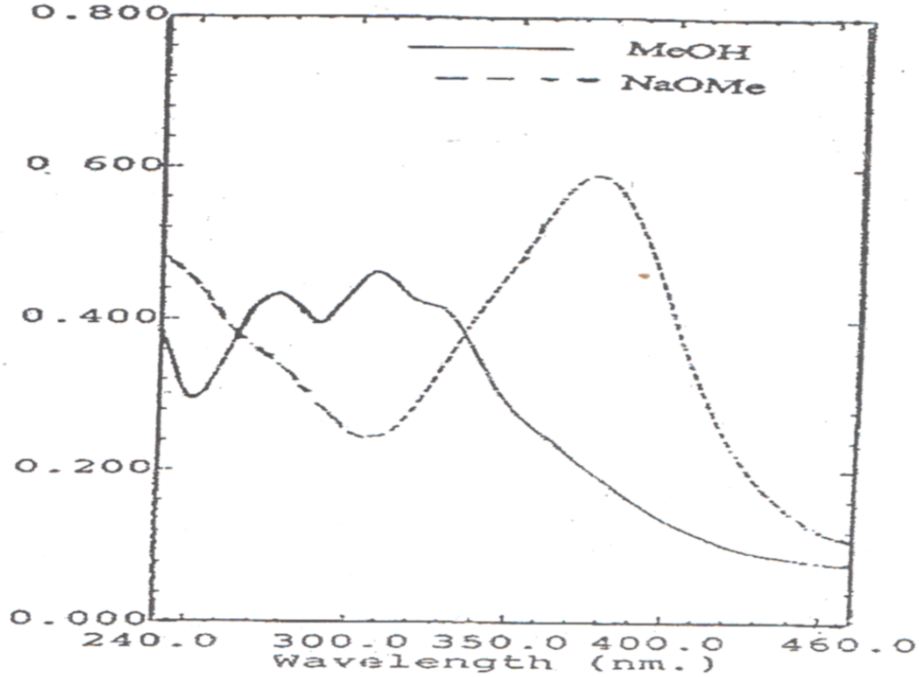

0.800
0 600
0.400
0.200
0.000
240.0
300.0
350.0
400.0
460.0
Wavelength (nm.)
MeOH
NaOMe

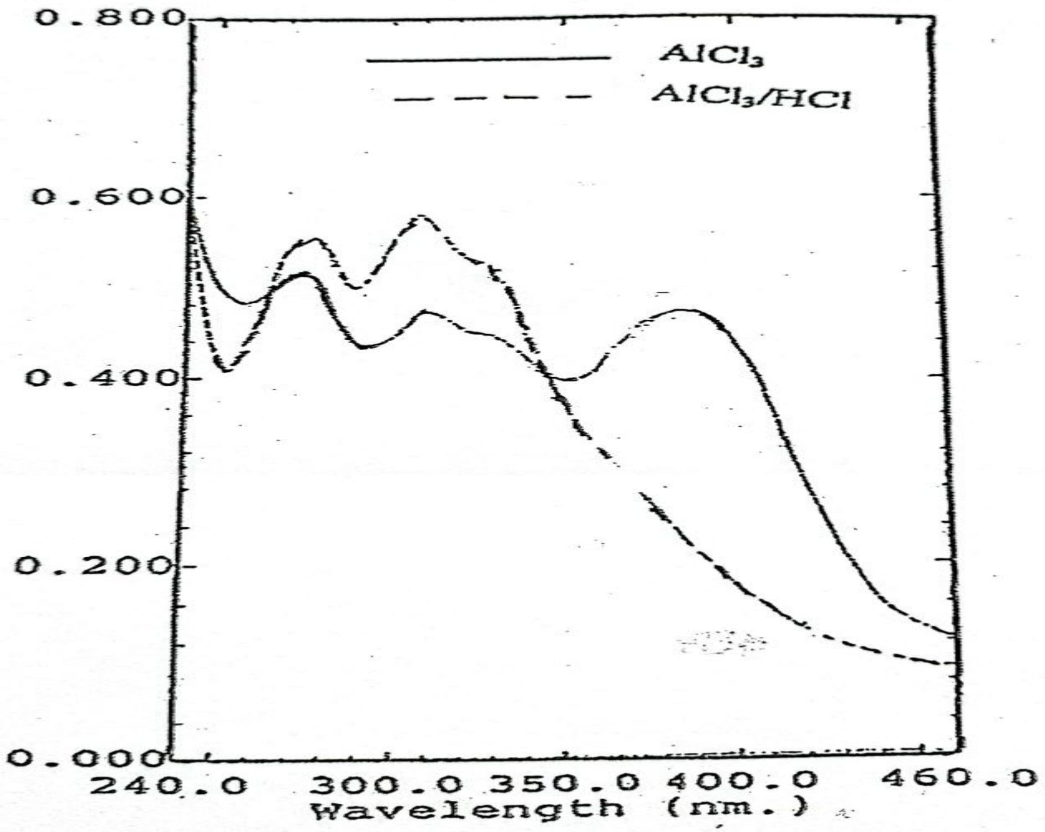
0.800
0.600
0.400
0.200
0.000
240.0
300.0
350.0
400.0
460.0
Wavelength (nm.)
AlCl3
AlCl3/HCl

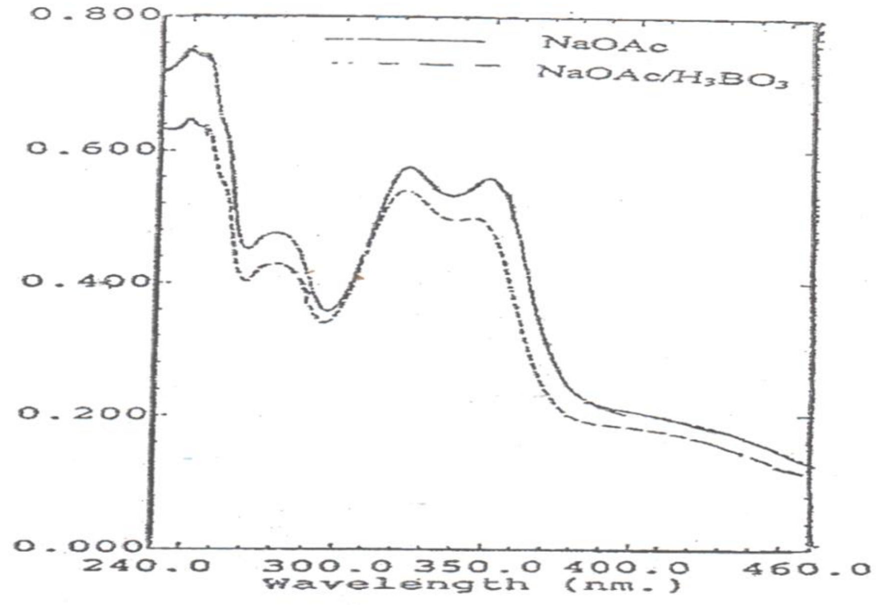

UV spectrum of compound 8

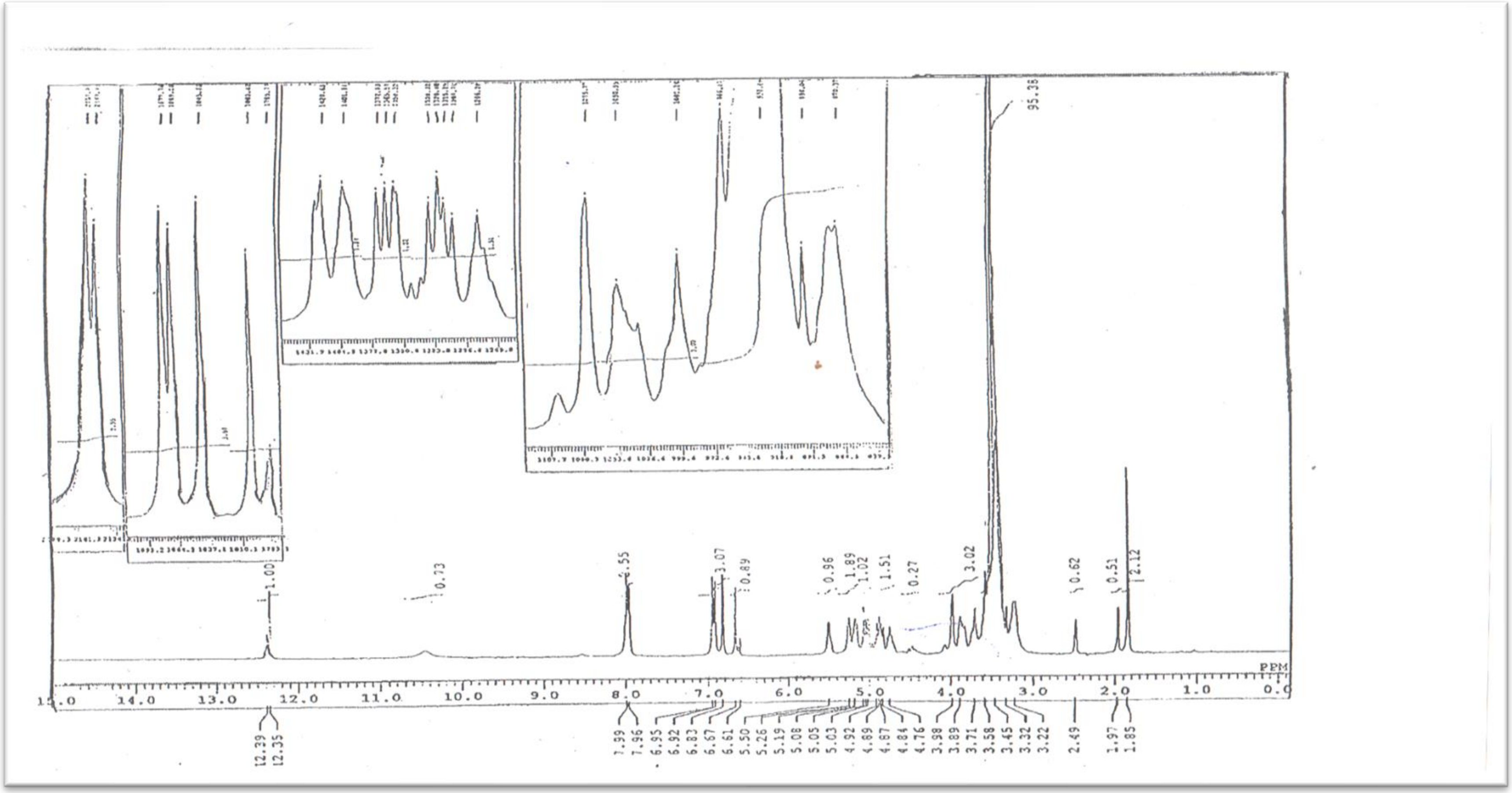

^{1}HNMR spectrum of compound 8 in DMSO –d6

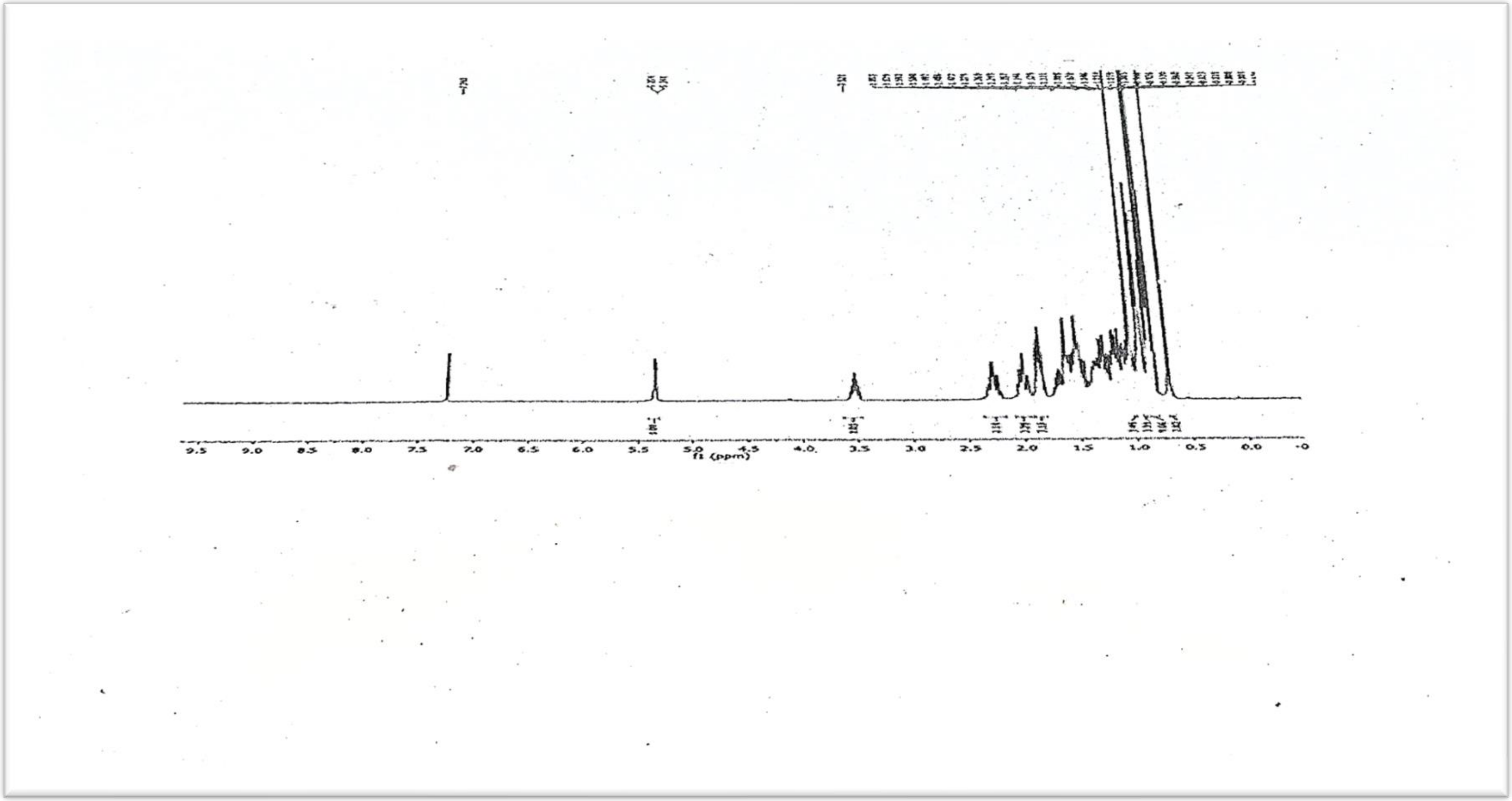

^{1}HNMR spectrum of compound 9 in $CDCl_3$

Materials and Methods

SI 3. Determination titers of viruses by plaque titration assay

Six-well tissue culture plates were seeded with Vero-E6 cells (10^5 cells/well). At 90–100% confluence (one-day post-seeding), cells were washed twice with 1X fetal bovine serum (FBS), the virus was diluted 10-fold in a medium with 2% FBS (Lonza, USA) and 2% antibiotic-antimycotic mixture (Lonza, Walkersville. MD, USA). 100 µL of each dilution was mixed with a 400 µL infection medium to inoculate one well of Vero-E6 cells. The plates were incubated at 37 °C with 5% CO_2 for 1 h. The wells were then aspirated to remove residual inoculum. Then, each well was immediately covered with 2 mL of overlay containing 1:1 of 2X medium and 2% agarose type 1 (Lonza, Basel, Switzerland). Plates were then incubated at 37 °C with 5% CO_2 for 3 days. The formation of the plaques was microscopically observed daily. Once clear plaques could be visualized, 1 mL of 10% formaldehyde was added to each well for 2 h at room temperature for cell fixation and virus inactivation. The formaldehyde was then discarded and the plates were rinsed with water and dried. For visualization of the plaques, 1 mL of the staining solution (1% crystal violet and 20% methanol in distilled water) was added to each well and incubated at room temperature for 5 min, the dye was then discarded as described before [1,2]. Viral plaques were then counted, and virus titer was calculated through the following equation:

Plaque forming unit (PFU)/mL = Number of plaques × virus dilution × volume of inoculum x multiplicand number to complete the inoculum volume to one mL.

SI 4. MTT cytotoxicity assay (CC_{50})

Samples were diluted with Dulbecco's Modified Eagle's Medium (DMEM). Stock solutions of the test compounds were prepared in 10 % DMSO in *dd* H_2O. The cytotoxic activity of the extracts was tested in Vero-E6 cells by using the 3-(4, 5-dimethylthiazol-2-yl)-2, 5-diphenyltetrazolium bromide (MTT) method with minor modification [3,4]. Briefly, the cells were seeded in 96 well-plates (100 µL/well at a density of 3×10^5 cells/mL) and incubated for 24 h at 37 ºC in 5% CO_2. After 24 h, cells were treated with various concentrations of the tested samples in triplicates. After a further 72 h, the supernatant was discarded and cell monolayers were washed with 1X sterile phosphate buffer saline (PBS) 3 times and MTT solution (20 µL of 5 mg/mL stock solution) was added to each well and incubated at 37 °C for 4 h

followed by medium aspiration. In each well, the formed formazan crystals were dissolved with 200 µL of DMSO (0.04 M HCl in absolute isopropanol = 0.073 mL HCl in 50 mL isopropanol). The absorbance of formazan solutions was measured at λmax 540 nm with 620 nm as a reference wavelength using a multi-well plate reader. The plot of % cytotoxicity versus sample concentration was used to calculate the concentration which exhibited 50% cytotoxicity (TC_{50}). The % of cytotoxicity compared to the untreated cells was determined with the following equation:

$$\% \, cytotoxicity = \frac{(absorbance \; of \; cells \; without \; treatment - absorbance \; of \; cells \; with \; treatment)X100}{absorbance \; of \; cells \; without \; treatment}$$

SI 5. Plaque reduction assay

The assay was carried out according to the method of Hayden *et al.* (1980) in a six-well plate where Vero-E6 cells (10^5 cells / mL) were cultivated for 24 h at 37 °C. The Middle East respiratory syndrome-related coronavirus isolate (NRCE-HKU270 (Accession Number: KJ477103.2) virus was diluted to give 10^3 PFU/well and mixed with the safe concentrations of the tested samples and incubated for 1 h at 37 °C before being added to the cells. The growth medium was removed from the cell culture plates and the cells were inoculated with (200 µL/well) virus with the tested samples and 300 µL of infection medium, After 1 h contact time for virus adsorption with shaking every 15 min after that remove inoculum and add safe concentrations of the tested samples were added with 3 mL of 2X DMEM supplemented with 2% agarose with 1:1 to cell monolayer, plates were left to solidify and incubated at 37 °C till the formation of viral plaques (3 to 4 days). Formalin (10%) was added for 2 h then plates were stained with 1% crystal violet in 80 mL distilled water and 20 mL methanol. Control wells were included where the untreated virus was incubated with Vero-E6 cells as discussed earlier [5,6]. Finally, plaques were counted and the percentage reduction in plaques formation in comparison to control wells was recorded as follows:

% inhibition= viral count (untreated) - viral count (treated)/viral count (untreated) x 100

SI 6. Mode of action of virus inhibition

The possible mode of action of virus inhibition by the selected plants' extracts was examined at three different stages of the virus propagation cycle and based on three main possible modes of action: (i) Inhibition of budding and viral replication. (ii) The ability of each extract to inhibit of attachment of the

virus to infected cells-membrane fusion known as blocking the viral entry (viral adsorption); and (iii) The direct effect of each extract to inactivate the virus viability (virucidal activity) [7,8]. Additionally, the above-mentioned mode of action could account for the recorded antiviral activities either independently, or in combinations. In this regard, the interaction between the selected plants' extracts and the MERS-CoV virus could be explained through the following three different modes of action:

SI 6.1. Viral replication

The viral replication assay was carried out according to Kuo *et al.* [9,10] in a 6-well plate where VERO-E6 cells were cultivated (10^5 cells/mL) for 24 h at 37 ^oC. The virus was diluted to 10^3 PFU/well and applied directly to the cells and incubated for 1 h at 37 ^oC. Unabsorbed viral particles were removed by washing cells 3 successive times with supplements free-medium. The extract was applied at different concentrations, and after 1 h contact time, add 3 mL of 2X DMEM medium supplemented with 2% agarose was to the cell monolayer. Plates were left to solidify and incubated at 37 ^oC till the appearance of viral plaques. Cell monolayers were fixed in 10% formalin solution for 2 h, and stained with crystal violet. Control wells were included where Vero-E6 cells were incubated with the virus and didn't treat with the extract. Finally, plaques were counted and the percentage reduction in plaque formation in comparison to control wells was recorded as above mentioned.

SI 6.2. Viral adsorption

Vero-E6 cells were cultivated in a 6 wells plate (10^5 cells/mL) for 24 h at 37 ^oC for the viral adsorption assay using the Zhang *et al.* method [11]. The plant extract was applied at different concentrations in a 200 µL medium without supplements and co-incubated with the cells for 2 h at 4 ^oC. The unabsorbed extract was removed by washing cells 3 successive times with supplements free-medium then the virus diluted was co-incubated with the pretreated cells for 1 h followed by adding 3 mL DMEM supplemented with 2% agarose. Plates were left to solidify and then incubated at 37 ^oC to allow the formation of viral plaques, fixed and stained as above mentioned to calculate the percentage reduction in plaques formation in comparison to control wells where untreated Vero-E6 cells were directly infected with the virus.

SI 6.3. Virucidal

The virucidal assay was carried out [12] in a 6 wells plate where Vero-E6 cells were cultivated (10^5 cells/mL) for 24 h at 37 ^oC. A volume of 200 µL serum-free DMEM containing virus was added to the

concentration of the tested extract. After 1 h incubation, the mixture was diluted using serum-free medium 3 times each 10-fold which still allows the existence of viral particles to grow on Vero-E6 cells but leaves nearly no extract and 100 µL of each dilution was added to the Vero-E6 cell monolayer. After 1 h contact time, the DMEM overlayer was added to the cell monolayer. Plates were left to solidify and then incubated at 37 ^{o}C to allow the formation of viral plaques, fixed and stained as above mentioned to calculate percentage reduction in plaques formation in comparison to control wells where cells were infected with the virus that was not pretreated with the tested extract.

SI 7. Inhibitory concentration 50 (IC_{50}) determination

In 96-well tissue culture plates, 2.4×104 Vero-E6 cells were distributed in each well and incubated overnight at a humidified 37 °C incubator under 5% CO_2 condition. The cell monolayers were then washed once with 1x PBS and subjected to virus adsorption (hCoV-19/Egypt/NRC-03/2020 (Accession Number on GSAID: EPI_ISL_430820)) for 1 h at room temperature. The cell monolayers were further overlaid with 100 µL of DMEM containing varying concentrations of the test compounds. Following incubation at 37 °C in a 5% CO2 incubator for 72 h, the cells were fixed with 100 µL of 4% paraformaldehyde for 20 min and stained with 0.1% crystal violet in distilled water for 15 min at RT. The crystal violet dye was then dissolved using 100 µL absolute methanol per well and the optical density of the color is measured at 570 nm using Anthos Zenyth 200rt plate reader (Anthos Labtec Instruments, Heerhugowaard, Netherlands) [13,14]. The IC_{50} of the compound is that required to reduce the virus-induced cytopathic effect (CPE) by 50%, relative to the virus control.

References

1. Kutkat, O.; Moatasim, Y.; Al-Karmalawy, A.A.; Abulkhair, H.S.; Gomaa, M.R.; El-Taweel, A.N.; Abo Shama, N.M.; GabAllah, M.; Mahmoud, D.B.; Kayali, G.; et al. Robust antiviral activity of commonly prescribed antidepressants against emerging coronaviruses: in vitro and in silico drug repurposing studies. *Scientific Reports* **2022**, *12*, 12920, doi:10.1038/s41598-022-17082-6.
2. Mahmoud, A.; Mostafa, A.; Al-Karmalawy, A.A.; Zidan, A.; Abulkhair, H.S.; Mahmoud, S.H.; Shehata, M.; Elhefnawi, M.M.; Ali, M.A. Telaprevir is a potential drug for repurposing against SARS-CoV-2: computational and in vitro studies. *Heliyon* **2021**, *7*, e07962.
3. Alnajjar, R.; Mostafa, A.; Kandeil, A.; Al-Karmalawy, A.A. Molecular docking, molecular dynamics, and in vitro studies reveal the potential of angiotensin II receptor blockers to inhibit the COVID-19 main protease. *Heliyon* **2020**, *6*, e05641.
4. Elmaaty, A.A.; Eldehna, W.M.; Khattab, M.; Kutkat, O.; Alnajjar, R.; El-Taweel, A.N.; Al-Rashood, S.T.; Abourehab, M.A.S.; Binjubair, F.A.; Saleh, M.A.; et al. Anticoagulants as Potential SARS-CoV-2 Mpro Inhibitors for COVID-19 Patients: In Vitro, Molecular Docking, Molecular Dynamics, DFT, and SAR Studies. *International Journal of Molecular Sciences* **2022**, *23*, 12235.
5. El-Masry, R.M.; Al-Karmalawy, A.A.; Alnajjar, R.; Mahmoud, S.H.; Mostafa, A.; Kadry, H.H.; Abou-Seri, S.M.; Taher, A.T. Newly synthesized series of oxoindole–oxadiazole conjugates as potential anti-SARS-CoV-2 agents: in silico and in vitro studies. *New Journal of Chemistry* **2022**, *46*, 5078-5090, doi:10.1039/D1NJ04816C.
6. Elebeedy, D.; Badawy, I.; Elmaaty, A.A.; Saleh, M.M.; Kandeil, A.; Ghanem, A.; Kutkat, O.; Alnajjar, R.; Abd El Maksoud, A.I.; Al-karmalawy, A.A. In vitro and computational insights revealing the potential inhibitory effect of Tanshinone IIA against influenza A virus. *Computers in Biology and Medicine* **2022**, *141*, 105149, doi:https://doi.org/10.1016/j.compbiomed.2021.105149.
7. Elebeedy, D.; Elkhatib, W.F.; Kandeil, A.; Ghanem, A.; Kutkat, O.; Alnajjar, R.; Saleh, M.A.; Abd El Maksoud, A.I.; Badawy, I.; Al-Karmalawy, A.A. Anti-SARS-CoV-2 activities of tanshinone IIA, carnosic acid, rosmarinic acid, salvianolic acid, baicalein, and glycyrrhetinic acid between computational and in vitro insights. *RSC Advances* **2021**, *11*, 29267-29286, doi:10.1039/D1RA05268C.
8. Kandeil, A.; Mostafa, A.; Kutkat, O.; Moatasim, Y.; Al-Karmalawy, A.A.; Rashad, A.A.; Kayed, A.E.; Kayed, A.E.; El-Shesheny, R.; Kayali, G.; et al. Bioactive Polyphenolic Compounds Showing Strong Antiviral Activities against Severe Acute Respiratory Syndrome Coronavirus 2. *Pathogens* **2021**, *10*, 758.
9. Kuo, Y.C.; Lin, L.C.; Tsai, W.J.; Chou, C.J.; Kung, S.H.; Ho, Y.H. Samarangenin B from Limonium sinense suppresses herpes simplex virus type 1 replication in Vero cells by regulation of viral macromolecular synthesis. *Antimicrobial agents and chemotherapy* **2002**, *46*, 2854-2864, doi:10.1128/aac.46.9.2854-2864.2002.
10. Soltane, R.; Chrouda, A.; Mostafa, A.; Al-Karmalawy, A.A.; Chouaïb, K.; dhahri, A.; Pashameah, R.A.; Alasiri, A.; Kutkat, O.; Shehata, M.; et al. Strong Inhibitory Activity and Action Modes of Synthetic Maslinic Acid Derivative on Highly Pathogenic Coronaviruses: COVID-19 Drug Candidate. *Pathogens* **2021**, *10*, 623.
11. Harcourt, J.; Tamin, A.; Lu, X.; Kamili, S.; Sakthivel, S.K.; Murray, J.; Queen, K.; Tao, Y.; Paden, C.R.; Zhang, J. Severe acute respiratory syndrome coronavirus 2 from patient with coronavirus disease, United States. *Emerging infectious diseases* **2020**, *26*, 1266.
12. Schuhmacher, A.; Reichling, J.; Schnitzler, P. Virucidal effect of peppermint oil on the enveloped viruses herpes simplex virus type 1 and type 2 in vitro. *Phytomedicine : international journal of phytotherapy and phytopharmacology* **2003**, *10*, 504-510, doi:10.1078/094471103322331467.

13. Aljuhani, A.; Ahmed, H.E.A.; Ihmaid, S.K.; Omar, A.M.; Althagfan, S.S.; Alahmadi, Y.M.; Ahmad, I.; Patel, H.; Ahmed, S.; Almikhlafi, M.A.; et al. In vitro and computational investigations of novel synthetic carboxamide-linked pyridopyrrolopyrimidines with potent activity as SARS-CoV-2-MPro inhibitors. *RSC Advances* **2022**, *12*, 26895-26907, doi:10.1039/D2RA04015H.
14. Elagawany, M.; Elmaaty, A.A.; Mostafa, A.; Abo Shama, N.M.; Santali, E.Y.; Elgendy, B.; Al-Karmalawy, A.A. Ligand-based design, synthesis, computational insights, and in vitro studies of novel N-(5-Nitrothiazol-2-yl)-carboxamido derivatives as potent inhibitors of SARS-CoV-2 main protease. *Journal of Enzyme Inhibition and Medicinal Chemistry* **2022**, *37*, 2112-2132, doi:10.1080/14756366.2022.2105322.
